# Anomalous Cosmic Rays and Heliospheric Energetic Particles

**DOI:** 10.1007/s11214-022-00890-7

**Published:** 2022-04-28

**Authors:** J. Giacalone, H. Fahr, H. Fichtner, V. Florinski, B. Heber, M. E. Hill, J. Kóta, R. A. Leske, M. S. Potgieter, J. S. Rankin

**Affiliations:** 1 Lunar & Planetary Laboratory, University of Arizona, Tucson, AZ 85721 USA; 2 Argelander-Institute of Astronomy, University of Bonn, Bonn, Germany; 3 Institut für Theoretische Physik, Ruhr-Universität, Bochum, Germany; 4 Center for Space Plasma and Aeronomic Research (CSPAR), University of Alabama in Huntsville, Huntsville, AL 35805 USA; 5 Institute for Experimental and Applied Physics, Christian-Albrechts University in Kiel, 24188 Kiel, Germany; 6 Applied Physics Laboratory, Laurel, MD 20723 USA; 7 California Institute of Technology, Pasadena, CA 91125 USA; 8 Department of Astrophysical Sciences, Princeton University, Princeton, NJ 08540 USA

**Keywords:** Particle acceleration, Cosmic rays, Shocks, Cosmic ray transport, Pickup ions, Heliosphere

## Abstract

We present a review of Anomalous Cosmic Rays (ACRs), including the history of their discovery and recent insights into their acceleration and transport in the heliosphere. We focus on a few selected topics including a discussion of mechanisms of their acceleration, escape from the heliosphere, their effects on the dynamics of the heliosheath, transport in the inner heliosphere, and their solar cycle dependence. A discussion concerning their name is also presented towards the end of the review. We note that much is known about ACRs and perhaps the term *Anomalous Cosmic Ray* is not particularly descriptive to a non specialist. We suggest that the more-general term: “Heliospheric Energetic Particles”, which is more descriptive, for which ACRs and other energetic particle species of heliospheric origin are subsets, might be more appropriate.

## Introduction

In the early 1970’s, analysis of the spectrum of galactic cosmic rays (GCR) revealed an unusual, or “anomalous”, enhancement at lower energies. In particular, the GCR intensity did not decrease with decreasing energy, as was expected based on our understanding that low-energy GCRs were unable to reach 1 AU due to their interaction with the solar wind. The anomalous enhancements were most notable in the spectra of oxygen and helium. A solar origin to these enhancements was not favored (Garcia-Munoz et al. 1973; Hovestadt et al. 1973; McDonald et al. 1974). These have since become known as anomalous cosmic rays (ACRs).

**Fig. 1:**
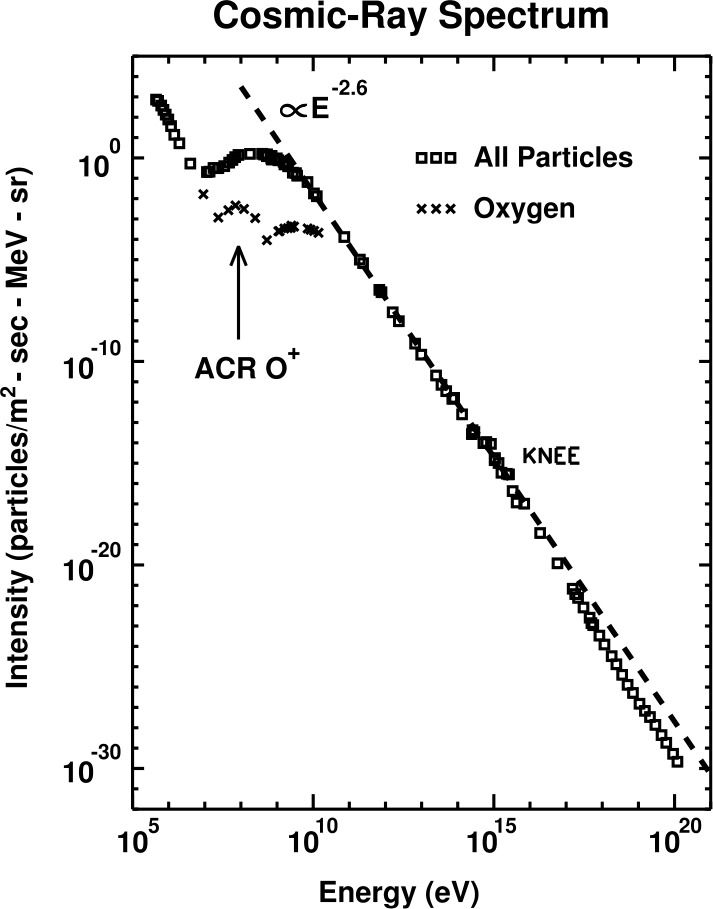
The cosmic-ray spectrum at 1 AU. This figure is from Giacalone et al. (2012), and originally from Jokipii (1990)

Figure 1 shows the full cosmic ray spectrum, including both GCRs and ACRs. GCRs are those indicated with the black squares, for which the spectrum has an approximately power law dependence on energy above a few GeV, and turns over, or is “modulated” at energies below about 1 GeV. This modulation is caused by the interaction of GCRs, most of which are produced in supernovae remnants very far form the Sun, with our heliosphere. Low energy ($\ll\text{GeV}$) GCRs have considerable difficulty reaching the inner heliosphere due to their interaction with the solar wind. ACRs are visible as an additional enhancement in the spectrum of oxygen (cross symbols), as indicated in the figure with the arrow, at about 100 MeV, which is a bit below the modulated part of the GCR oxygen spectrum. ACRs are enhancements in the spectrum of helium, nitrogen, oxygen, neon, protons, but not in carbon (Klecker 1995). The intensity of ACRs were observed to increase with distance from the Sun, suggesting their source is in the distant heliosphere (McDonald et al. 1974).

It was recognized soon after their discovery, by Fisk et al. (1974), that ACRs are accelerated interstellar pickup ions. These are interstellar atoms which have become ionized through either charge exchange with the solar wind, or photo-ionization by solar UV. Interstellar atoms enter the solar system because the Sun moves relative to the local interstellar medium (e.g. Frisch 1996). Upon ionization, a pickup ion is subject to forces which cause it to drift away from the Sun towards the outer heliosphere. Moreover, a freshly ionized pickup ion has an energy of about $\sim1~\text{keV}$ relative to a frame of reference moving with the solar wind and must be significantly accelerated to achieve the energy associated with ACRs. It was first noted by Pesses et al. (1981) that this energization could be achieved by the interaction with the solar wind termination shock. The acceleration mechanism, in this case, is known as diffusive shock acceleration discovered in the late 1970’s (Axford et al. 1977; Krymsky 1977; Bell 1978; Blandford and Ostriker 1978). This theory is based on solutions to the well-known cosmic-ray transport equation (Parker 1965) for the distribution function of particles interacting with a shock-like plasma discontinuity. Jokipii (1986) solved this equation giving the first quantitative calculation for the distribution of ACRs in the heliosphere, including acceleration at the termination shock and transport in the heliosphere.

## Summary of Some Key Historical Observations

### Energy Dependent Evolution of Energy Spectrum and Intensity Across the Termination Shock

Prior to the Voyager crossings of the termination shock, ACRs were observed to have large, positive radial gradients, indicating the source was more distant (McDonald et al. 1974). It was also noted that the low-energy end of ACR (H and He) spectra revealed a large increase with radial distance compared to the higher energy end (Stone et al. 1999). Assuming that the particles diffuse from the outer heliosphere, this observation suggests the lower-energy particles have a smaller diffusion coefficient than the higher energy ones.

It was also expected that the energy spectrum would become a power law, at least up to the typical ACR energy of several tens to about 200 MeV (total energy), at the time of the crossing of the termination shock because it was thought that the shock was the source of the particles (e.g. Pesses et al. 1981). This was the prediction of the models (e.g. Jokipii 1986; Potgieter and Moraal 1988; Steenberg and Moraal 1996). However, neither Voyager observed the expected power-law energy spectrum upon crossing the termination shock, and instead observed that in the 3–30 MeV energy range, the spectrum did not increase at the shock, remaining “modulated” (Stone et al. 2005, 2008). Later, when each spacecraft was deeper into the heliosheath, the spectrum eventually filled in with a nearly power law dependence on energy with a spectral index of about −1.6 (Cummings et al. 2008). Clearly, the particles above a few MeV were not accelerated locally where each spacecraft crossed the shock. Instead, their fluxes continued to rise as the spacecraft moved into the heliosheath. This suggests they are accelerated by some other mechanism than shock acceleration, or are accelerated elsewhere on the shock. This is discussed further below.

In contrast, at energies below a few MeV, both Voyagers observed significant enhancements of the particle flux at the time of the shock crossing (Decker et al. 2005, 2008). This was found to be consistent with the acceleration of pickup ions locally at the termination shock (Giacalone and Decker 2010; see also Florinski et al. 2009).

### Characteristics of Termination Shock Particles

As noted in the previous section, the term “TSP” refers to particles whose flux was observed to peak locally at the crossing of the termination shock (Decker et al. 2005, 2008), and were also observed to increase in intensity for a few years before the shock crossings (see Fig. 13). Unusual TSP anisotropies were observed by V1 in 2001–2003, prior to the shock crossing. These anisotropies were directed away from the Sun and were nearly aligned with the Parker spiral magnetic field. At the time, this was thought to be unusual because it suggested a source inside the location of the spacecraft, and not beyond it. In fact, it was suggested that V1 had crossed the termination shock (Krimigis et al. 2003), which, at the time, was assumed to be roughly spherically shaped. However, it was soon realized that the observations could be understood if the surface of the shock was blunt shaped, while the Parker spiral magnetic field lines were very nearly perpendicular to the radial direction (Jokipii et al. 2004). A key prediction of this model was that when V2 crossed the shock, the anisotropies would be observed to be moving in the opposite direction (sunward) since the V2 crossing would be on the other side of the sub-solar point of the blunt-shaped shock compared to V1. This was indeed observed (Decker et al. 2005, 2008), confirming the picture put forth by Jokipii et al. (2004). The current paradigm is that the termination shock is blunt shaped. This is also seen in large-scale fluid simulations of the heliosphere (e.g. Zank 1999).

TSPs were also observed to be highly variable with intermittent increases in intensity. It was suggested that these variations, and the significant field-aligned anisotropies, could be understood as the spacecraft crossing through magnetic field lines that were either filled or devoid of energetic particles depending on the magnetic connectivity to the source (assumed to be the termination shock) (Giacalone and Jokipii 2006). In addition, it was also observed that some of the intermittent intensity enhancements occurred at times of specific magnetic polarity reversals associated with crossings of the heliospheric current sheet (HCS) (Richardson et al. 2006). This was interpreted by Giacalone and Burgess (2010) as the result of the interaction and acceleration of pickup ions at the point of intersection of the termination shock and HCS of a particular polarity such that the accelerated particles drift upstream, away from the shock, along the HCS.

In the heliosheath, particles below an MeV or so, were observed to be remarkably uniform in intensity as seen by both Voyagers 1 and 2, despite the large difference in their trajectories leading to a $>100~\text{AU}$ separation. At the heliopause crossing, their intensity dropped to background levels, indicating their rapid escape from the heliosphere at this point (Krimigis et al. 2019). This is discussed further in Sect. 4. Regarding the uniform intensity in the heliosheath, this suggests that their acceleration at the shock is also uniform along the shock surface. This was supported by numerical simulation of pickup ion acceleration at the termination shock at multiple locations (Giacalone et al. 2021).

### ACR Charge States

Analysis of observations of ACRs revealed a rather low charge state. Klecker et al. (1980) reported an upper bound for ACR oxygen O of 4, and later, Adams et al. (1991) found that the observations were consistent with a charge state of 1. ACRs with higher charge states have been reported as well, as discussed below, but it is now generally understood that most ACRs below 100–200 MeV total energy are singly charged. Adams and Leising (1991) noted that a singly charged ACR moving in the interstellar medium will lose additional electrons if the source is beyond about 0.2 pc. This can also be thought of as a constraint on the age to be less than about four years. This places a rather stringent constraint on the time scale associated with the acceleration of pickup ions to the observed ACR energies. Jokipii (1992) noted that this time scale could be achieved most naturally for acceleration at the nearly perpendicular termination shock, and other mechanisms were too slow.

Multiply charged ACRs have also been observed (Mewaldt et al. 1996). This work suggests an even more-stringent constraint of the acceleration time scale of about one year. Large-scale global modeling of ACRs, including acceleration at the termination shock, transport in the heliosphere, and further stripping of electrons via interaction with the background plasma using known formulas for the cross-sections, by Jokipii (1996) found that higher-energy ACRs, $>300~\text{MeV}$, change from being singly charged to multiply charged. This modeling, which agreed well with the observations, was expanded upon by Barghouty et al. (2000) using updated hydrogen-impact ionization rates giving an even better agreement with the observations.

An important key takeaway from the historical observations of ACR charge states is that the acceleration time scale must be of the order of a year. In a separate study, analysis of Pioneer-10 observations showed that there was a shift in the correlation between ACR-fluxes with Lyman-alpha data (a proxy for solar variability) that was attributed to the time it takes to accelerate pickup ions to the observed ACR energies (Scherer et al. 1997). The time scale was found to be less than a year, which is consistent with the charge-state observations. Given that the initial energy of the particles is of the order of a keV, the energy of a freshly ionized pickup ion, there must be a nearly 5 order-of-magnitude-increase in the particle energy on a time scale of a year.

### Recovery of ACR Intensity Following a Global Merged Interaction Region: Pinpointing the ACR Source Location

In the early 1990’s, a large global merged interaction region (GMIR), a large magnetic/plasma disturbance associated with intense solar activity, moved through the heliosphere causing a depletion in the intensities of GCRs and ACRs (McDonald et al. 2000; Webber et al. 2001). The reduction of the GCR intensity is the well-known Forbush-decrease phenomenon. McDonald et al. (2000) noted that the recovery time of the flux of ACRs and GCRs were different, with the ACRs recovering considerably more rapidly than the GCRs. This suggests that the ACR source is nearer to the spacecraft than is the GCR source. In fact, these authors used the recovery time to estimate the distance to the source, coming up with a location of the termination shock between 80–96 AU. This is consistent with the distances at which each Voyager later crossed the termination shock. Moreover, time-dependent simulations of ACRs and GCRs during the passage of a GMIR through the outer heliosphere by Jokipii and Kota (2001), see also le Roux and Fichtner (1999), obtained results that were in agreement with the observations. These calculations assumed an ACR source at the termination shock.

## Acceleration Mechanisms

### Acceleration at the Termination Shock

#### General Considerations: Diffusive Shock Acceleration

Particle acceleration at shocks has been studied for many years, with a major breakthrough in understanding occurring in the late 1970’s with the development of diffusive shock acceleration (DSA) theory (Axford et al. 1977; Bell 1978; Blandford and Ostriker 1978; Krymsky 1977). Perri et al, this journal, also addresses the current state of knowledge of this topic and this topic was also recently reviewed by Liu and Jokipii (2021). The theory is based on the fact that charged particles are accelerated by any compression of the plasma, and shocks provide a very strong plasma compression owing to their the change in plasma density over a scale that is of the order of the thermal ion inertial length. In DSA theory, the rate of energy gain depends on how effectively the particles are trapped near the shock, through their diffusion in turbulent magnetic fields, which are either pre-existing (independent of the shock), or associated with plasma instabilities related to the shock.

The fundamental equation of DSA theory is known as the Parker equation (Parker 1965), which is also used in cosmic-ray transport theory. The equation gives the phase-space distribution function, $f$, as a function of position vector $x_{i}$, momentum magnitude, $p$ and time, $t$. It is given by:

1 $$ {\frac{\partial f }{\partial t}} = { \frac{\partial }{\partial x_{i}}} \left [\kappa _{ij} { \frac{\partial f }{\partial x_{j}}}\right ] - U_{i} { \frac{\partial f }{\partial x_{i}}} + {\frac{1 }{3}} { \frac{\partial U_{i} }{\partial x_{i}}} \left [ { \frac{\partial f }{\partial \ln p}} \right ] + Q $$

where $\kappa _{ij}$ is the diffusion tensor which contains components associated with spatial diffusion both along and across the magnetic field (symmetric components), as well as large-scale guiding center drifts (the anti-symmetric components), $U_{i} = {\mathbf{U}}$ is the plasma velocity, and $Q$ is a source. The term that gives acceleration, or deceleration, is the second from the last, and one notes immediately that it is proportional to the divergence of the flow speed, which from the continuity equation in fluid dynamics, is related to the plasma compression. There are two key assumptions in the derivation of this equation, one is that the particle distribution is assumed to be isotropic in pitch angle, and the other is that the electric field, $\mathbf{E}$ is that of ideal MHD, given by ${\mathbf{E}}=-(1/c){\mathbf{U}}\times {\mathbf{B}}$, where $B$ is the magnetic field vector, and $c$ is the speed of light. Equation (1) contains the four main transport effects: advection, spatial diffusion, drifts, and energy change.

DSA is essentially a solution to Equation (1), for the case in which the flow velocity vector changes instantaneously at a shock. This is discussed in Giacalone et al. (2012). In steady state, it is found that the downstream distribution function has a power-law dependence on the momentum with a spectral index that depends only on the ratio of the upstream to downstream plasma density. Moreover, the distribution upstream decreases with distance from the shock, exponentially if the diffusion coefficient does not depend on distance. It is constant downstream of the shock, in the steady-state case. We note from Fig. 13 that from about the year 2000 until the crossing of the termination shock by each Voyager, the intensity of 140–220 keV ions increased until the shock crossing and then became constant downstream, as expected from the theory. Of course, as is now well known, the peak was not at the shock at higher energies (ACRs), which is discussed further below.

For the case of the termination shock, the magnetic field is essentially normal to the unit normal to the shock everywhere over the shock’s surface owing to its quasi-spherical shape and because the Parker spiral is highly wound in the outer heliosphere. This is also true at each of the heliographic poles if one considers the behavior of the transverse and radial components of the turbulent magnetic field, as suggested by Jokipii and Kota (1989). They noted that while the radial component of the interplanetary magnetic field behaves as $1/r^{2}$, the transverse component falls off only as $1/r$. Thus, even if the transverse field is small compared to the radial component near the Sun, at the termination shock, nearly 100 AU from the Sun, the transverse field dominates. Directly above the poles, this depends somewhat on the choice of parameters for the turbulent fluctuations, but the affected portion of the termination shock is quite small, less than 0.01% of the surface. Thus, the field is almost entirely in the transverse direction at the termination shock, even at the heliographic poles. In this case, particles gain energy primary by drifting along the shock due to the gradient in the magnetic field, in the direction of the motional electric field. In the absence of particle diffusion, this process is known as shock-drift acceleration (Armstrong et al. 1985). This process is actually a subset of DSA, since drift along the shock, and energy gain through drift along the electric field, is contained within DSA theory.

For the time-dependent case, and also for the case in which there are losses due to geometrical considerations (such a finite-sized shock), the power-law part of the distribution is only at the lowest energies. The characteristic energy where the spectrum deviates from a power-law depends on a number of factors, such as the age of the shock, the size and geometry of the shock, and the rate of acceleration, among other things. For the case of the termination shock, ignoring solar-cycle related effects, the problem is essentially time independent, but the shock is of finite size. In fact, it was emphasized by Jokipii and Giacalone (1998) that the maximum energy is limited by the finite drift distance along the shock. It was noted that the electric potential difference from pole-to-equator is about 240 MeV/charge, which, if fully realized for protons, would give a maximum energy near to the observed maximum ACR energy. The departure from the power law at the shock, and the maximum energy, can also be understood as the energy at which the rate of acceleration at the shock becomes comparable to the rate of energy loss in the expanding solar wind (note that the last term in Equation (1) gives energy loss for the solar wind). Potgieter and Moraal (1988) emphasized the importance of shock curvature, noting that the power-law result was obtained only for a planar shock and the spectral rollover occurs at the energy for which the relevant diffusive length scale is of the same order as the radius of curvature of the shock. The topic of the spectral rollover was also addressed by Steenberg and Moraal (1999).

#### Acceleration at the Blunt-Shaped Termination Shock

It is now widely accepted that the termination shock is not spherically shaped, even on average, as was assumed in the early models of cosmic ray acceleration and transport, but, instead, has a blunt shape. This shape arises because of the flow of the interstellar plasma, providing a dynamic pressure that is highest in the region where the interstellar flow velocity is opposite to that of the solar wind. This is the so-called “nose” region, and at this point, the termination shock is closer to the Sun than it is at other locations. The blunt-shaped termination shock was first suggested by analysis of TSP anisotropies (Jokipii et al. 2004). Although not emphasized earlier, it can also be seen in large-scale MHD simulations of the heliosphere (e.g. Zank 1999).

The blunt-shaped termination shock has a significant and important effect on the acceleration of particles. While the shock normal varies with longitude, the Parker spiral magnetic field is essentially normal to the radial direction. Thus, as was noted by McComas and Schwadron (2006), this leads to the situation in which the intersection point of any given magnetic line of force with the shock moves along the shock from its first crossing, at the nose, towards the flanks of the heliosphere. Since it takes time to accelerate particles, only the lowest-energy particles, accelerated the most rapidly, are expected to peak at this point. It takes up to a year to accelerate to the highest energies, during which time the intersection point will have moved far towards the flanks of the heliosphere. Thus, one expects that at the flanks is where the ACRs will have the highest intensity.

**Fig. 2:**
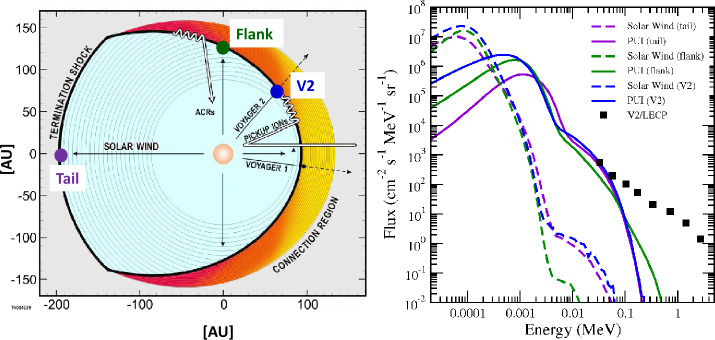
(Left panel) Representation of the blunt-shaped termination shock, interplanetary magnetic field lines, and ACR intensity, adapted from McComas and Schwadron (2006) to include three locations along the termination shock. (Right panel) energy spectra of pickup ions (solid curves) and solar wind (dashed curves) at the three locations shown in the left panel, with the same color representation. This figure is taken from Giacalone et al. (2021). The black squares in the right figure are Voyager 2 LECP observations taken just after the crossing of the termination shock (see also Giacalone and Decker 2010, for more details)

The conceptual, intuitive picture (shown in the left panel of Fig. 2, discussed below) suggested by McComas and Schwadron (2006) has been quantitatively confirmed by Kóta and Jokipii (2008) and Schwadron et al. (2008). Moreover, it provides a natural explanation for the observations. For instance, at low energies, the TSPs were observed to peak at the shock and subsequently had a relatively constant intensity. In contrast, the highest-energy particles, the ACRs, continued to rise into the heliosheath which is also consistent with the picture above. This is because when the peak fluxes are in the flanks, the particles subsequently are transported within the heliosheath. Along the trajectory of the Voyagers, the peak will not be at the shock, but farther into the heliosheath, as observed (cf. McComas et al. 2019). Moreover, analysis of ACR anisotropies by Cummings et al. (2019), showing a diffusive streaming of ACRs coming from the flanks of the heliosphere support this picture.

It should also be noted that similar effects may arise for the case in which the shock interacts with large-scale plasma turbulence. In this case, the shock surface may become rippled, which can effect the particle acceleration at the termination shock (e.g. Li and Zank 2006). If the scale of the ripples is larger than the characteristic scale associated with the particle diffusion, effects similar to those discussed above for the blunt-shock case arise (e.g. Kóta 2010; Guo et al. 2010). Similarly, even at a planar shock, the turbulent plasma upstream of the shock may lead to meandering magnetic lines of force which also produce similar effects as considered by Kóta and Jokipii (2008). It is likely that these effects also play an important role in the acceleration of heliospheric energetic particles, such as TSPs and ACRs, at the termination shock.

#### Acceleration at Low Energies: The Injection Problem

We know that ACRs and TSPs originate as interstellar pickup ions whose energy upon their creation is of the order of $\sim1~\text{keV}$. It is well accepted that DSA theory is applicable for particles with energies much larger than this, but it is also applicable at lower energies, such as those associated with pickup ions, provided there is sufficient scattering to maintain an isotropic distribution and, also, to remain near the shock. It is now generally known that shocks accelerate low energy particles, even a fraction of those that are part of the thermal distribution, to high energies (Ellison and Eichler 1984; Scholer 1990; Giacalone et al. 1992; Giacalone 2005b). The question arises of how this can happen given that their random velocity is similar to the bulk speed of the plasma which advects them downstream. Since shocks convert energy entering the shock – in the form of dynamic pressure – into thermal energy, the (bulk) plasma is subsonic downstream of the shock. Thus, a fraction of the downstream plasma can return to the shock and enter back into the upstream flow. Thus, even shock-heated thermal plasma is capable of participating in the shock-acceleration process, and at the termination shock, the dominant species is shock-heated interstellar pickup ions. Accelerated particles likely play a critical role in the energy dissipation at shocks, and the efficiency of the acceleration depends on how much of the incident energy at the shock can be proportioned to the high-energy tail.

For the case of interest here, i.e. pickup ion acceleration at the termination shock, one must also consider that the average magnetic field is nearly perpendicular to the shock normal, even for the blunt-shaped shock. It has been previously thought that such shocks have a difficulty to accelerate low-energy ions (e.g. Ellison et al. 1995). However, it has been shown that such shocks are indeed capable of accelerating low (and even thermal) energy particles provided there is a sufficient level of pre-existing magnetic fluctuations present in the plasma that passes through the shock (e.g. Giacalone 2005b). Also, as can clearly be seen in Fig. 13, the peak intensity of 140–220 keV ions, admittedly higher energy than freshly ionized pickup ions, but still much lower than ACR energies, is at the shock. Recently, Lario et al. (2019) analysed observations of energetic particles at interplanetary shocks, including observations which include the thermal, suprathermal, and high-energy tail of the distribution, and found that very low-energy ions are accelerated directly at the shock, even including at least one event in which the shock-normal angle was close to ninety degrees. Neergaard-Parker and Zank (2012) and Neergaard-Parker et al. (2014) quantified this further by assuming a form for source distribution of particles in diffusive shock acceleration, and fit the shock-accelerated spectrum to that observed for a number of interplanetary shocks. They found that in many cases, acceleration of solar-wind, at near thermal energies, accounted for the intensity of the observed high-energy tail. Thus, observations of interplanetary shocks, as well as that at the termination shock, generally reveal that there is no problem accelerating low energy ions at quasi-perpendicular shocks. Thus, it is reasonable to say that it is a misconception to suggest otherwise.

Hybrid simulations of the interaction of pickup ions with the termination shock, showing efficient acceleration, were performed initially by Giacalone and Decker (2010), and more recently by Giacalone et al. (2021). The simulations treat all the ions (solar wind and pickup) kinetically and effectively solve for the kinetic shock physics on ion scales. It was found that the pickup ions are thermalized at the shock, and a high-energy tail forms from the shock-heated distribution. This high-energy tail has a nearly power-law dependence on energy up to about 50–100 keV, where the simulated spectrum falls off sharply. This rapid fall off of the spectrum is caused by the limitations imposed by the very small simulation domain and finite simulation time. By comparing the results with V2/LECP observations, and by using simulation parameters consistent with the observed plasma and field parameters, it was found that at about 50 keV, the flux of accelerated pickup ions agreed well with the observed TSP intensities downstream of the shock. This suggests strongly that TSPs are, in fact, accelerated interstellar pickup ions, representing a key step in the process which ultimately leads to ACRs. More-recently, Zirnstein et al. (2021) performed a similar study using a test-particle approach of particle acceleration at the termination shock in the presence of magnetic turbulence, consistent with that observed by Voyager 2. The results were compared to IBEX observations of energetic neutral atoms. It was found that a high-energy, power-law tail formed from the pickup ion distribution due to shock acceleration, with a spectral index consistent with that inferred from IBEX observations.

Recently, Giacalone et al. (2021) extended the study of Giacalone and Decker (2010) to study the acceleration of pickup ions at other locations along the termination shock in addition to that where V2 crossed. The right panel of Fig. 2 is taken from this paper. The left panel shows a representation of blunt-shaped termination shock (thick black line) and representative Parker-spiral magnetic field lines (gray), and a color-code representation of the ACR intensity with red being the highest and yellow the lowest. Three locations along the shock are identified in the left panel with purple, green, and blue circle symbols. This figure is an adaptation of the figure shown in McComas and Schwadron (2006). The locations identified in this figure relate to the hybrid simulated spectra, in the same colors, in the right panel. In the right panel, solid lines are hybrid-simulation pickup ion differential intensity spectra as a function of energy, and dashed lines are those for the solar wind.

Figure 2 also shows V2/LECP data just after the crossing of the termination shock (black square symbols). The simulated spectra at about 50–100 keV agree well with the observations. Above this energy, the simulated spectra fall off dramatically, which is due to limits imposed by the use of the small simulation domain and finite simulation time, and are not comparable to the observations at these energies. As noted above, the fact that the simulated spectra match the observations at 50–100 keV indicates that TSPs are accelerated pickup ions. This figure also indicates that there is little variation of the 50–100 keV intensity at widely different locations along the termination shock. Given the TSPs were observed to be rather uniform throughout the heliosheath, prior to their going away completely at the heliopause, and that they are uniform along the shock as well, suggests that TSPs are likely quite uniform throughout the heliosheath. For more details, see Giacalone et al. (2021).

With regards to the physics of particle acceleration at low energis, the primary concern is how particles are trapped near the shock. Thermal particles, or those just slightly more energetic than the bulk plasma, are largely dominated by convection. However, as the plasma is heated across the shock, some of these particles are capable of returning upstream. For shocks that move normal to the magnetic field, the particles must move across the mean magnetic field in order to stay near the shock. This has led to the suggestion that nearly-perpendicular shocks have difficulty in accelerating low-energy particles. However, magnetic field-line meandering, caused by large-scale turbulence in the solar wind, is one means by which low-energy ions, even those near the thermal population, can remain near the shock and be efficiently accelerated (cf. Giacalone 2005a,b). Is has also been suggested that the required process for the injection of particles into DSA can also be related to the reflection of ions at the shock (Chalov and Fahr 1996; Dworsky and Fahr 2000; le Roux et al. 2000). Verscharen and Fahr (2008) and Fahr and Verscharen (2008) investigated ion reflections from the shock due to pitch-angle scattering driven by Alfvènic turbulence and demonstrated that suprathermal pick-up ions are not necessary, but that the injection already works for the energetic part of the regular thermal solar wind ions. This process works especially effectively for parallel MHD shock configurations where about 18% of the incoming solar wind ions are reflected back to the upstream side and do enter the ACR acceleration process. This pitch-angle-induced ion reflection could in principle continue working even further downstream of the shock since Alfvénic turbulence amplitudes grow downstream (Zank et al., this journal).

### Acceleration Mechanisms in the Heliosheath

The topic of the acceleration of cosmic rays in the inner heliosheath received attention after the Voyager spacecraft crossed the termination shock. As noted above, they found that only the intensities of low-energy particles peaked at the shock, while those of the ACRs increased further in the downstream region and that their spectra exhibited multiple power-law spectral slopes (Decker et al. 2005; Cummings et al. 2006; Cummings and Stone 2013). At the time, this was not well understood since classical DSA models were not readily applicable (le Roux and Fichtner 1997).

Explanations for this unexpected behaviour include refined DSA models (as discussed above) (McComas and Schwadron 2006; le Roux and Webb 2009; Giacalone and Decker 2010; Senanayake et al. 2015), second-order Fermi processes such as momentum diffusion (Moraal et al. 2006; Ferreira et al. 2007; Fisk and Gloeckler 2009; Kallenbach et al. 2009; Strauss et al. 2010a), and magnetic reconnection (Lazarian and Opher 2009; Drake et al. 2010; Zhao et al. 2019). A discussion of these mechanisms in the context of ACRs was included in the comprehensive review by Giacalone et al. (2012). While, so far, there is still some debate regarding the acceleration mechanism of ACRs, there is agreement with regard to the seed population, which is the pick-up ion population.

A further acceleration of the seed population of ACRs in the inner heliosheath is also of interest in the context of energetic neutral atoms (ENAs). The main ENA component observed by the IBEX spacecraft is produced via charge exchange of solar wind and pick-up protons with interstellar neutral atoms in this region (e.g., Siewert et al. 2013). ENAs 1 keV–6 keV need a pre-acceleration of protons, which is usually assumed to be momentum diffusion (Fahr et al. 2016; Zirnstein et al. 2018). For such modeling, kappa distributions have been considered, which are discussed in the chapter by Perri et al. in this book. For ENAs with higher energies, as observed with SoHO/HSTOF, charge exchange with ACRs has been considered (Czechowski et al. 2001) but without taking into account their acceleration in the inner heliosheath. This modeling is, therefore, left as a task for the future.

#### Numerical Modeling of Anomalous Oxygen in the Heliosheath

Strauss et al. (2010b) developed a numerical model for the acceleration and propagation of anomalous Oxygen in the heliosheath. Apart from diffusive shock acceleration at the termination shock (TS), their model also included momentum diffusion and adiabatic energy changes in the heliosheath (see also Langner et al. 2006; Ferreira et al. 2007). The TS compression ratio and particle injection efficiency were obtained from appropriate hydrodynamic models (Scherer et al. 2006). Their model satisfactorily reproduced the modulated spectrum of anomalous Oxygen observed at the TS and the unfolding into the heliosheath. They concluded that a combination of momentum diffusion and adiabatic energy increase, under reasonable assumptions of the solar wind speed in the heliosheath, produce a feasible continuous acceleration process to explain the main features of the full anomalous Oxygen spectrum in the heliosheath.

Strauss et al. (2010a) emphasized that the observed anomalous Oxygen spectrum near the TS reveals a power law form above the energy roll-over point, instead of an exponential spectral cut-off at energies $>20~\text{MeV/nuc}$ (Webber et al. 2007). Using the numerical model mentioned above, they showed that this deviation from an expected exponential form could be explained taking into account the acceleration of multiply-charged anomalous Oxygen up to 70 MeV/nuc above which Galactic Oxygen dominates. They illustrated how the roll-over energy shifts to higher energies when the higher charge states for anomalous Oxygen were incorporated into their simulations, while the low energy portion of these spectra (1–10 MeV/nuc) remains relatively unaltered. This causes in the process an increase in high energy anomalous Oxygen intensities throughout the heliosphere, also at the Earth. This was displayed in the modulated spectra at the Earth in comparison with relevant observations near the Earth from several space missions (Marsden et al. 1999; Hill et al. 2002; Mewaldt 2006; Cummings et al. 2009). The topic of multiply-charged anomalous cosmic rays was discussed previously in this chapter (see Jokipii 1996; Mewaldt et al. 1996; Mewaldt 2006).

**Fig. 3:**
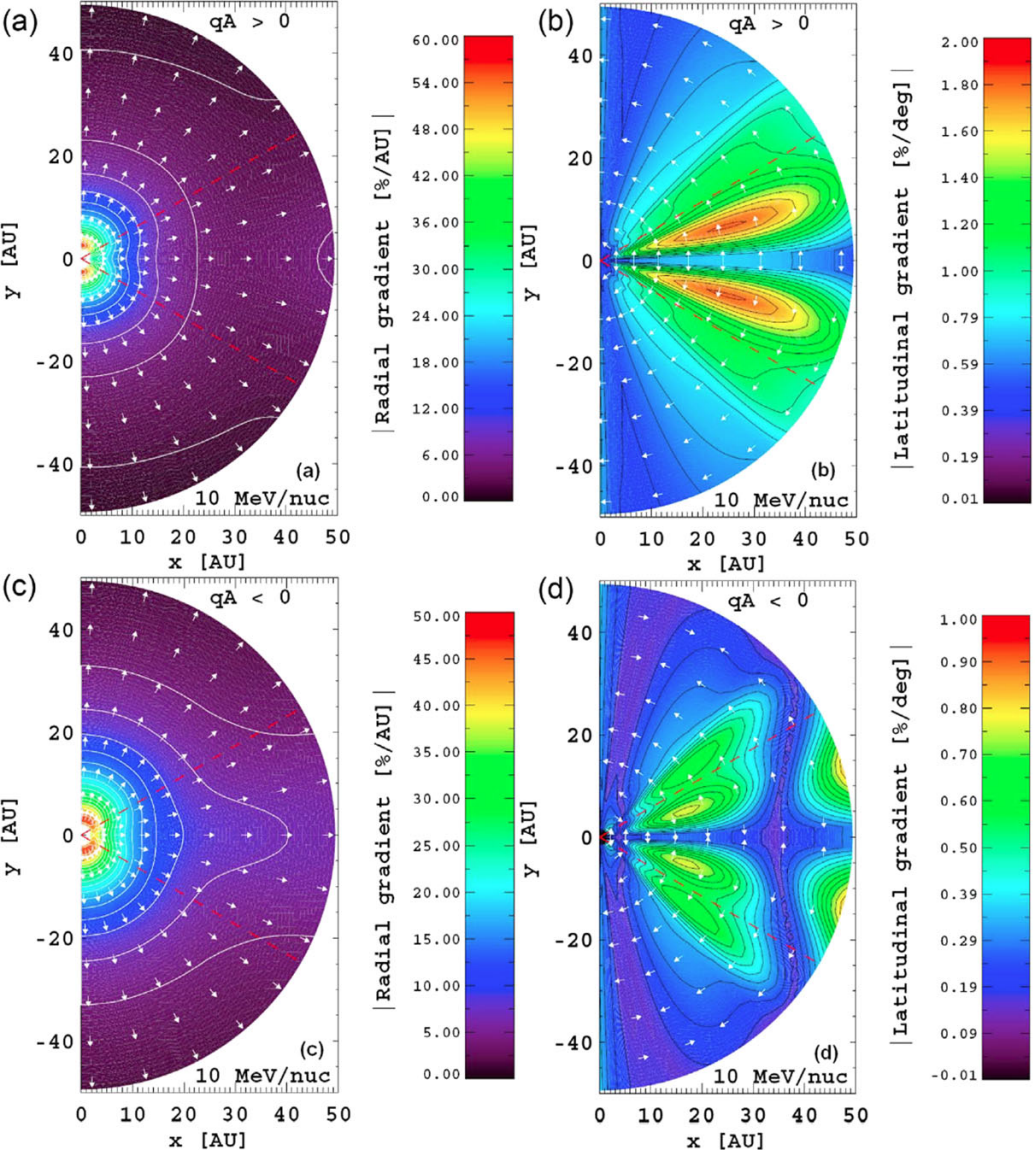
Simulated global spatial gradients for 10 MeV/nuc anomalous Oxygen shown in the meridional plane in the inner heliosphere for the two HMF ‘polarity’ cycles; panels (**a**, **b**) for $A>0$ and panels (**c**, **d**) for $A<0$. Colour scale indicates the value of the radial gradient in panels (**a**, **b**) and the value of the latitudinal gradient in panels (**c**, **d**), whereas the arrows indicate the direction of increasing intensity. Dashed red lines in each panel indicate the latitudinal extent of the heliospheric current sheet. (Fig. 10 from Strauss and Potgieter 2010)

Strauss and Potgieter (2010) modeled ACR Oxygen spectra and associated spatial gradients applicable to the inner heliosphere. They specifically simulated latitudinal and radial gradients during both heliospheric magnetic field polarity cycles and compared their simulations with observations close to and at the Earth; see their Table 1 for a list of observed and radial gradients. Their global simulation of spatial gradients for anomalous Oxygen is depicted in Fig. 3. This model was a refinement of the numerical model of Strauss et al. (2010b), applicable to the main features of this type of charged particle population which was not addressed earlier.

## Transport Within the Heliosheath and Escape from the Heliosphere

The V1 and V2 heliopause (HP) encounters revealed that the magnetic boundary of the solar system was not impervious to HEPs (heliospheric energetic particles, as discussed in Sect. 8). Viewed on a global scale, the HP appeared to be essentially a tangential discontinuity characterized by an approximately twofold increase in the magnetic field strength without a significant change in direction. The situation was much more complex on short ($<\text{AU}$) scales. V1 encountered two narrow regions within the last 0.3 AU before the heliopause where the magnetic field was stronger, and the ACR intensity was depressed by a factor of 3–5 compared with the surrounding heliosheath (Krimigis et al. 2013). The intensities dropped again at the HP itself. The decay time of these particles was rather different at the V1 and V2 crossings. In the case of the V1 crossing, the ACRs were seen to deplete over a time period of several days, but at V2 ACRs persisted for a month or more after the crossing. The extent of this boundary layer was found to be weakly dependent on particle’s velocity, but strongly rigidity-dependent (Stone et al. 2013). In addition, ions streaming along the field disappeared first, while those gyrating near the $90^{\circ }$ pitch angle persisted the longest. As a result, within the dropouts and the LISM boundary layer, ACRs developed a “pancake” distribution with a large second-order anisotropy.

The dropouts were interpreted by Krimigis et al. (2013) as interstellar magnetic flux tubes penetrating the HP, into the heliosheath, as a consequence of the magnetic interchange instability. The stability criteria were derived theoretically by Florinski (2015). The instability is driven by a force exerted on the magnetic field by ions moving along curved trajectories corresponding to the convexity of the HP, and requires a negative pressure gradient across the boundary. Because interchange is inhibited by a degree of magnetic rotation across the HP, it was suggested that a flux tube would undergo a kink and thus become aligned with the field on the opposite side.

**Fig. 4:**
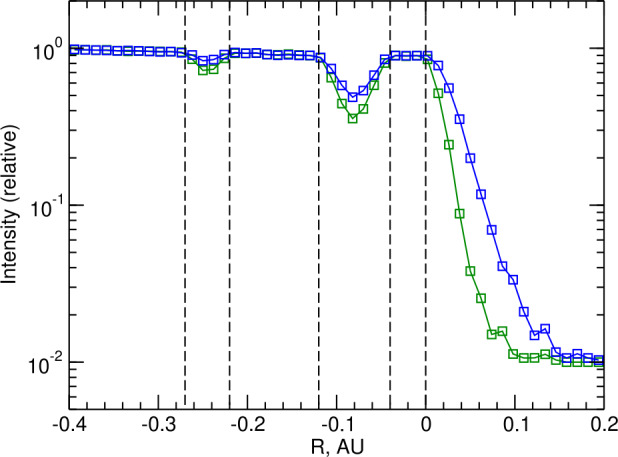
Model-derived spatial profiles of 5 MeV protons near the heliopause. The green and blue symbols refer to $\mu =1$ and $\mu =0$ particle populations. The flux tube crossing events and the heliopause itself are marked with vertical dashed lines. Figure reproduced from Florinski et al. (2015)

Florinski et al. (2013) simulated the anisotropies of 5 MeV protons near the HP by treating it as a shear layer with a large decrease in the amplitudes of magnetic fluctuations from the heliospheric to the interstellar side. They assumed that magnetic shear suppressed field line meandering across the HP, so that only particles with large pitch angle were able to cross into the VLISM (very local interstellar medium) by virtue of their large gyro-radius, whereas particles streaming parallel to the local field remained trapped inside the heliosheath. The model successfully explained the observed $\sim 5~\mbox{MeV}$ ion spatial profiles and pitch-angle distributions reported by Krimigis et al. (2013). These ideas were further developed by Florinski et al. (2015) who investigated the rigidity dependence of the ACR depletion layer. They proposed that gradient magnetic-field drift was responsible for spatially separating particles according to their rigidity because the gradient drift velocity is proportional to the gyro-radius. This mechanism requires an increase in the field strength toward the south, which is expected based on the magnetic field draping pattern from large-scale MHD simulations (e.g., Izmodenov and Alexashov 2020). Figure 4 compares simulated intensities of 5-MeV protons at two different pitch angles. Note, in particular, a more gradual decrease in particles gyrating near the $90^{\circ }$ pitch angle compared with those streaming parallel to the field lines. An alternative to this model was developed by Strauss and Fichtner (2014), who suggested that perpendicular diffusion was large at $90^{\circ }$ and decreased to zero as the pitch-angle cosine, $\mu \to \pm 1$. Subsequently, a theory was developed in support of this scenario. The theory predicted that perpendicular diffusion in the presence of compressive longitudinal fluctuations would indeed have the required dependence on pitch angle (Strauss et al. 2016).

Measuring the anisotropy of ACRs associated with the gradient in the density of their guiding centers, that was very prominent within the dropouts, allowed Florinski et al. (2015) to derive an independent estimate of the radial component of the plasma velocity in the heliosheath, on the heliospheric side of the HP. This observation was possible owing to the relative orientation of the multiple Voyager LET telescopes. The value for the radial speed obtained was consistent with zero or slightly positive, but under 5 km/s. This result is consistent with the notion that the HP is a tangential discontinuity, impervious to the low-energy solar-wind ions.

**Fig. 5:**
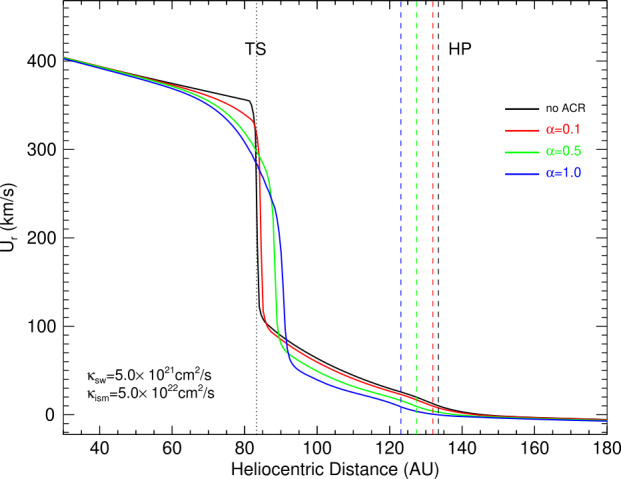
Simulated radial plasma speed along the Voyager 1 direction for three different PUI injection efficiencies $\alpha =0.1$, 0.5, and 1.0. The position of the heliopause is shown with vertical dashed lines. Figure reproduced from Guo et al. (2019)

Nonetheless, a sizable fraction of the higher energy ions were evidently able to overcome the magnetic shear layer and escape into the VLISM. Note that Gloeckler and Fisk (2016) concluded that the sum of the pressures of the solar wind, pickup ions, ACRs, plus the magnetic pressure on the heliospheric side of the HP exceeds the combined pressures of the interstellar plasma and magnetic field, which appears to contradict the notion of a pressure-balanced boundary. This difficulty is naturally resolved by adding the escaped heliospheric ions to the pressure balance on the interstellar side. Guo et al. (2018, 2019) investigated the effects of this pressure loss on the size of the heliosphere. In their model, the energetic particles were treated as a massless fluid whose energy density $E_{c}$ satisfied the equation

2 $$ \frac{\partial E_{c}}{\partial t}+\nabla \cdot [(E_{c}+p_{c}) \mathbf{u}-\kappa \nabla E_{c}]=\mathbf{u}\cdot \nabla p_{c}-\alpha p_{c} \nabla \cdot \mathbf{u}, $$

(e.g., Zank et al. 1993) where $p_{c}$ is the energetic particle pressure, $u$ is the plasma velocity, $\kappa $ is the energy-averaged diffusion coefficient, and $\alpha $ is the rate of injection from the low-energy pickup ion population that is not distinguished from the thermal plasma. It was found that the ACRs play a significant role in the structure of the outer heliosphere in some situations, being largely dependent on the momentum and energy transfer from the solar wind plasma to the ACRs, the diffusive shock acceleration at the termination shock, and the subsequent loss of ACRs across the heliopause and their rapid escape into the interstellar medium. Under favorable conditions, characterized by a large fraction of energy conversion $\alpha $ and a high enough diffusion coefficient $\kappa $ in the solar wind, the ACRs were found to reduce the width of the heliosheath by up to $\sim18~\text{AU}$, as seen in Fig. 5. Consequently, these results indicate that the effect of ACRs is a potential key factor for the global structure of the outer heliosphere in a numerical model that could partially explain the thickness of the heliosheath based on the timing of the HP and termination shock encounters of the two Voyager probes.

## Solar Cycle Dependence

**Fig. 6:**
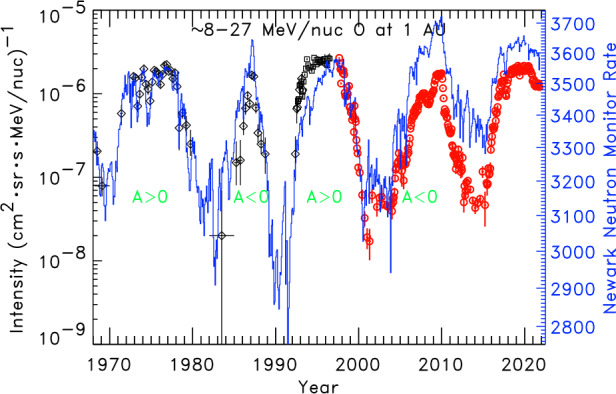
Intensities of $\sim8\text{--}27~\text{MeV/nucleon}$ oxygen measured at 1 AU during solar quiet periods since 1970 (data points; left axis) compared with the Newark neutron monitor count rate (blue; right axis) averaged over Bartels rotations. Red data points are from the ACE/SIS instrument, while black symbols are earlier published space-based measurements (see Mewaldt et al. 1993 for data references). Figure updated from Leske et al. (2013)

Observations of both ACRs and GCRs near Earth span multiple solar cycles. By 1 AU, both ACR and GCR intensities have been heavily modulated because of their transport in the solar wind, by an amount which varies greatly with the phase, and to some extent, the magnetic polarity of the solar cycle. As seen in Fig. 6, during periods of low sunspot number, solar minimum, and when the solar magnetic field points outward in the northern hemisphere ($A>0$), the intensities of positively-charged ACR and GCR ions tend to plateau for several years, creating a “flat-topped” profile. For the alternate magnetic polarity (still during solar minimum), when the field points outward in the southern hemisphere ($A<0$), the highest intensities persist for a much briefer period. This behavior is understood to arise from the fact that during $A<0$ solar minima, particles drift into the inner heliosphere along the heliospheric current sheet (HCS), while under $A>0$ conditions particles enter from the polar regions of the heliosphere and drift outward along the HCS (Jokipii and Thomas 1981; Kota and Jokipii 1983). The result is that in addition to the well-known 11-year cosmic-ray cycle pattern, there is also a 22-year cosmic ray cycle which results from the swapping of the solar magnetic polarity with consecutive solar minima.

During the 2008–2009 $A<0$ solar minimum period, cosmic-ray modulation was significantly less and GCR intensities were correspondingly higher than were seen in earlier cycles (Mewaldt et al. 2010); however, the ACR intensities remained below their 1996 values (Leske et al. 2013). The solar wind density, dynamic pressure and magnetic field strength were all quite weak (McComas et al. 2008; Smith and Balogh 2008). Yet, the HCS, which might be expected to be quite flat during the weakest part of the solar cycle, remained tilted. It only became “flattened” in 2009. This lead to speculation that since the ACR source intensity in the outer heliosphere is expected to be stronger at low heliographic latitudes during $A<0$ minima periods (Jokipii 1986; Florinski et al. 2004), while the GCRs are more uniformly distributed at the boundary of the heliosphere, that perhaps ACR intensities at 1 AU in near ecliptic plane (at Earth) were more sensitive to the higher HCS tilt angle than GCRs and thus not as greatly enhanced (Leske et al. 2013). Effects of the HCS tilt angle would be expected to be less important during the present $A>0$ cycle, however.

**Fig. 7:**
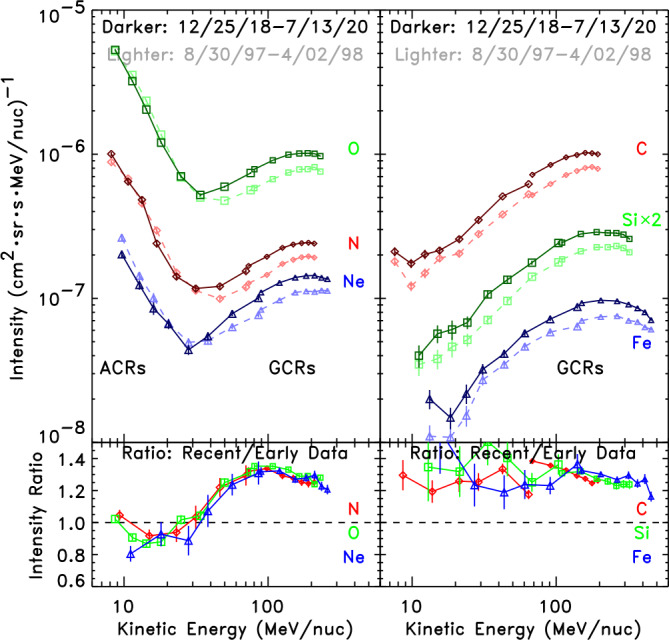
Energy spectra from the SIS (at lower energies) and CRIS (at higher energies) instruments on ACE for elements N, O, and Ne with strong ACR components (left panels) and C, Si, and Fe that are essentially pure GCRs at 1 AU (right panels). For each element, spectra are shown in two periods, one near the 1997 $A>0$ solar minimum (lighter dashed curves) and one at the 2018–2020 $A>0$ solar minimum (darker solid curves), with ratios of the spectra at the two time periods shown in the bottom panels. All GCRs are seen to be significantly enhanced by a similar amount in the latest solar minimum relative to the earlier minimum throughout this energy range, while ACRs are somewhat depleted. For a similar comparison of spectra in the 2009 $A<0$ minimum with those in the 1997 $A>0$ period, see Leske et al. (2013)

Although GCR intensities in the last two solar minima have been higher than previously observed, the same is not the case for the ACR intensities. Note from Fig. 6 that the ACR intensities are seen to return to similar peak intensities at successive solar maxima. Differences between the ACR and GCR behavior for different ion species and energies in the last two $A>0$ solar minima are seen in more detail in the spectra shown in Fig. 7. The intensities of all GCR elements from C to Fe were enhanced by $\sim30\%$ in 2020 over their values in 1997 at energies from at least 50 to over 200 MeV/nucleon. For elements that do not have a strong ACR component, such as those in the right panels of Fig. 7, this enhancement extends all the way down to 10 MeV/nucleon. In contrast, the ACR elements N, O, and Ne all show no change or even a slight depletion in their 2020 peak intensities compared with their 1997 values, as seen in the lower left panel of Fig. 7. This is clearly not simply an energy-dependent effect on the spectra since it is not present in GCRs (right panel at the same energies), nor can it be attributed to instrumental effects since the ACRs and GCRs were measured with the same instrument.

Both ACRs and GCRs arrive at 1 AU from the outer heliosphere and are subject to similar transport, and subsequent modulation, since they move in the same field and plasma. They generally have the same waxing and waning with the solar cycle, as indicated by the similarities in their intensity variations as the solar cycle progresses (e.g. Fig. 6). However, the ACRs are “locally” accelerated, as described in the previous sections of this review, while the GCRs are not, and come from far away from the heliosphere. The GCR behavior shows that there must be less modulation in the current solar minimum period compared to the last $A>0$ minimum, but the ACR intensities seem rather unaffected. This is not presently well understood. On the one hand, the GCR intensity varied considerably across the HP (see Rankin, this journal), suggesting that perhaps much of the GCR modulation occurs beyond the source of the ACRs. On the other hand, the production of ACRs depends on their acceleration rate, which depends on the solar magnetic field, shock strength, and other factors, and these likely play a critical role. This is discussed further below. Moreover, the low-energy source of the ACRs, interstellar pickup ions, may have a lower intensity due to reduction in the ionization rate of inflowing interstellar neutral atoms because of the reduced solar output in the recent weak solar maxima (Sokół et al. 2019). At present, there is no widely accepted explanation for the observed differences in the ACR and GCR intensity variations (at Earth) with the solar cycle.

In addition to modifying the ACR intensity, changes in the ACR source might alter their composition and/or energy spectra. For example, the production of pick-up ions depends on both charge exchange collisions with the solar wind and photoionization by solar UV radiation. To ionize neutral Ne, photoionization is about 100 times more significant than charge exchange, while for oxygen the two processes contribute nearly equally (Sokół et al. 2019). Thus, if the solar wind density and speed, which governs charge exchange, varies by a different amount than the solar UV emission that determines the photoionization rate, the elemental composition of the pick-up ions and hence ACRs would be expected to change. Alternatively, if the ACR acceleration process is mass-dependent, changes in the plasma and fields near where ACRs are accelerated might affect the resulting composition of the ACRs. Similarly, reduced acceleration efficiency might preferentially deplete the higher energies, resulting in a softer spectrum. Changes in diffusion might alter the production of higher ionic charge states via stripping of the accelerated particles (Jokipii 1996), again changing the measured elemental spectra since the ions with higher charge states are observed at higher energies (Mewaldt et al. 1996; Klecker et al. 1997; Selesnick et al. 1997). These hypothetical composition and spectral changes would be much more subtle and harder to detect at 1 AU than the differences in the overall ACR intensity, but perhaps limits to these effects could be obtained from the existing data to help constrain models.

A reasonable explanation for the observed discrepancy in ACR-GCR solar-cycle variations is the potentially less effective acceleration of ACRs at the TS, as noted above. ACRs reaching Earth must originate from higher-energy ACRs at the TS because they lose energy during their transport to Earth; thus the observed ACR flux at Earth is sensitive to the flux at the high-energy part of the source spectrum at the TS. Moraal and Stoker (2010) were the first to point out that while the likely larger diffusion coefficient responsible for milder (or weaker) modulation will, at the same time, also lead to a slower rate of acceleration and a lower cut-off energy of the ACR source spectrum. This is because the rate of acceleration is inversely proportional to the diffusion coefficient $\kappa $ (e.g. Drury 1983). Note also that it can be shown that for the simplest spherical configuration, the power-law exponent associated with acceleration of particles at the spherical TS, takes the form: $\gamma = (3 V_{1}/\Delta V)[1 + 2 (V_{2}/\Delta V) (\kappa /(V_{1}R_{sh}))]$. The first term is the result from the usual planar-shock case, while the second term arises because of the spherical geometry, and inclusion of adiabatic cooling. The net result is that the spectrum steepens if either $\kappa $ increases or the shock radius, $R_{sh}$ decreases. The later may be related to the effects of varying solar-wind dynamic pressure.

Finally, the weaker heliospheric magnetic field results in faster particle drift along the shock front, which again leads to less effective acceleration. ACRs drift along the shock face as they are accelerated, hence the maximum energy of accelerated ACRs should be in the range of the electric potential difference between the heliospheric equator and pole, as we discussed previously (e.g. Jokipii 1996). This electric potential difference is determined by the 26 day rotational velocity of the Sun multiplied by the total open flux of the solar magnetic field, hence a weaker field should lower the maximum energy of ACRs.

At present we cannot single out the precise cause of the difference in solar-cycle behavior of the ACRs and GCRs. The mechanisms discussed above can, and likely do, act together, the exploration of their respective contribution needs further research.

## Radial and Latitudinal ACR Gradients in the Heliosphere

**Fig. 8:**
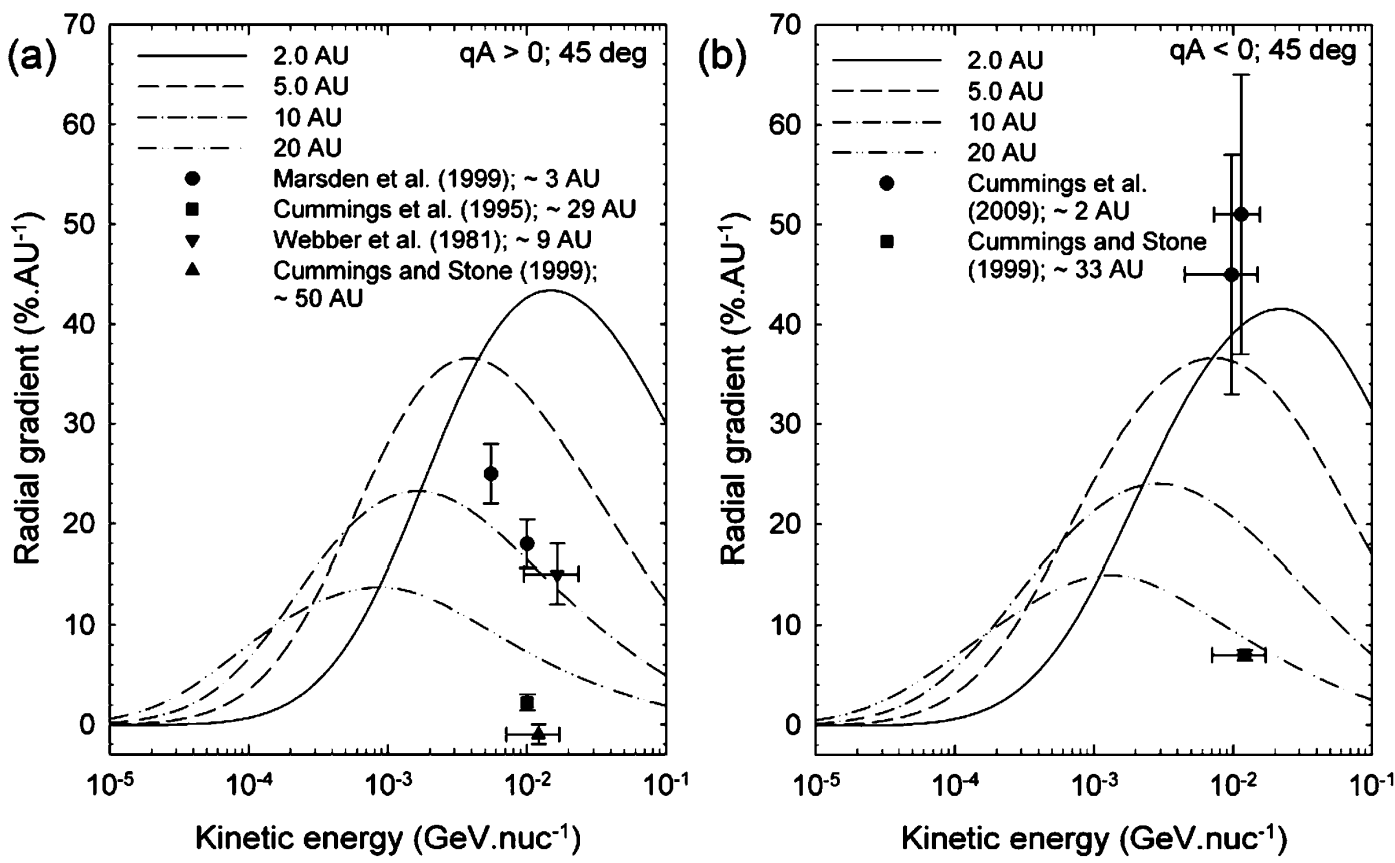
Simulated radial gradients at 2, 5, 10 and 20 AU and polar angle of 45 degrees for anomalous Oxygen for the $A>0$ magnetic cycle in panel (**a**) and for $A<0$ in panel (**b**). For comparison, observations of non-local radial gradient are shown as indicated. (Fig. 5 from Strauss and Potgieter 2010)

An key prediction from cosmic-ray transport models is that radial and latitudinal gradients of ACRs depend strongly on the solar magnetic cycle. The simulated radial gradients using the model described in the previous section at 2, 5, 10 and 20 AU, and a polar angle of 45 degrees, for anomalous Oxygen for $A>0$ and $A<0$ magnetic cycles are shown in Fig. 8. For comparison, observations of non-local radial gradient are shown as indicated. The value of 45%/AU for the radial gradient of anomalous Oxygen close and inside 1 AU were found by Marquardt et al. (2018) to be qualitatively consistent with the predictions from the model.

Observational evidence for positive and negative latitudinal gradients came from the two Pioneer and Voyager missions in the outer heliosphere (McKibben et al. 1979; Cummings et al. 1987; Christon et al. 1986). McKibben et al. (1979) and Cummings et al. (1987) reported values for the latitudinal gradients ranging from 2.1 to $3.1\%/^{\circ }$ and −2.2 to $-1.6\%/^{\circ }$ for ACR Helium at about 15 MeV/nuc. during an $A>0$- and $A<0$-solar magnetic cycles, respectively. For ACR oxygen Cummings et al. (1987) found even larger values for the latitudinal gradient (−3.7 to $-2.9\%/^{\circ }$). However their analysis was restricted to heliographic latitudes of less than $30^{\circ }$.

**Fig. 9:**
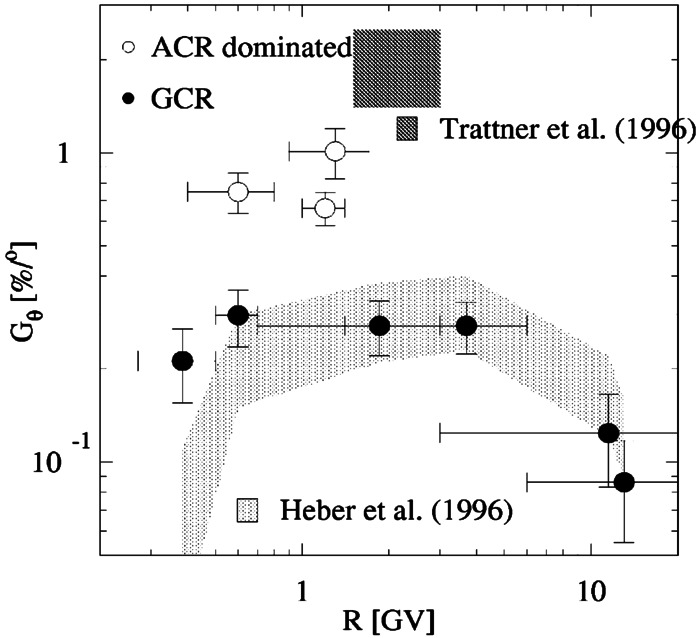
Overview of latitudinal gradients observed during the first and second Ulysses fast latitude scan

The Ulysses spacecraft mission provided instruments (Simpson et al. 1992; Klecker et al. 1993; Simpson et al. 1995; Lanzerotti and Maclennan 1995; Keppler et al. 1992) with which to measure cosmic ray latitudinal gradients in the heliographic polar regions. Trattner et al. (1995b,a, 1996), Lanzerotti and Maclennan (1995) and Heber et al. (1999) report latitudinal gradients varying between $0.39\%/^{\circ }$ to a $2.12\%/^{\circ}$ in an $A>0$-solar magnetic epoch. Figure 9, which comes from Heber (2001), displays the latitudinal gradient as a function of rigidity for both ACRs and GCRs during Ulysses’ first latitudinal scan. It was also found that the ACR oxygen gradient during the $A<0$ magnetic cycle was 5 times smaller than during the $A>0$-solar magnetic cycle (Cummings et al. 2009). These authors concluded that ACRs do not propagate into the inner heliosphere by drifting along the HCS. In contrast, latitudinal gradients in the outer heliosphere during the $A<0$-solar magnetic epoch are generally considerably larger, indicating that the transport of ACRs is dominated in the inner heliosphere by diffusion, rather than drift along the HCS. Simpson et al. (1996) also reported a north-south asymmetry in the cosmic rays observations from Ulysses after it had observed both polar regions of the Sun. They noted that while the latitudinal gradients in each hemisphere were quite similar in each hemisphere, and the modulation was approximately symmetric, the plane of symmetry was offset southward, by about 10 degrees from the heliographic equator. To our knowledge, this has not been fully explained.

## Observations of Anomalous Cosmic Rays Inside of 1 AU

The study of cosmic rays in the near-Sun environment is a territory that is just beginning to be explored. The first measurements from $\sim0.3$ to 1.0 AU were taken by the Helios spacecraft over 45 years ago, with no follow-up until Parker Solar Probe. Re-visiting the Helios data, Marquardt et al. (2018) investigated the spatial distribution of ACR oxygen during the 1974–1977 solar minimum and found a significant radial gradient: $48\pm12\%~\text{AU}^{-1}$ for energies of 9 to 29 MeV/nuc, roughly 3 times larger than measured by Pioneer 10 from 1 to 10 AU around the same time period. For instance, Webber et al. (1981) found values of $15\pm3\%~\text{AU}^{-1}$ from 1 to 10 AU for 9.5 to 24 MeV/nuc. These findings were not well reproduced by models. For example the model of Strauss and Potgieter (2010) for ACR oxygen in the heliosphere, including close to 1 AU, predicted the largest radial gradient is about 2 AU beyond the orbit of Earth, suggesting a smaller gradient inside 1 AU.

**Fig. 10:**
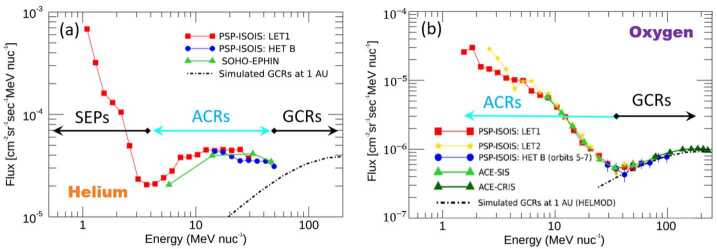
Event-subtracted spectra for Helium (**a**) and Oxygen (**b**) observed by several telescopes (LET1, red; LET2, yellow; HET B, blue) of the ISOIS instrument onboard Parker Solar Probe (PSP). 1 AU baseline measurements by (**a**) the EPHIN instrument on SOHO (SOHO-EPHIN; light green triangles), and (**b**) the SIS and CRIS instruments on ACE (light and dark green triangles, respectively) were used to correct for modulation due to long-term variations in solar output. Data were taken during the $\sim2018.7$ to $\sim2019.9$ and $\sim2018.7$ to $\sim2021.2$ time-frames, respectively. GCRs at 1 AU were simulated using the HelMod online calculator (www.helmod.org; see text for further details). Figure adapted from Rankin et al. (2021) (Helium) and Rankin et al. (in press) (Oxygen)

**Fig. 11:**
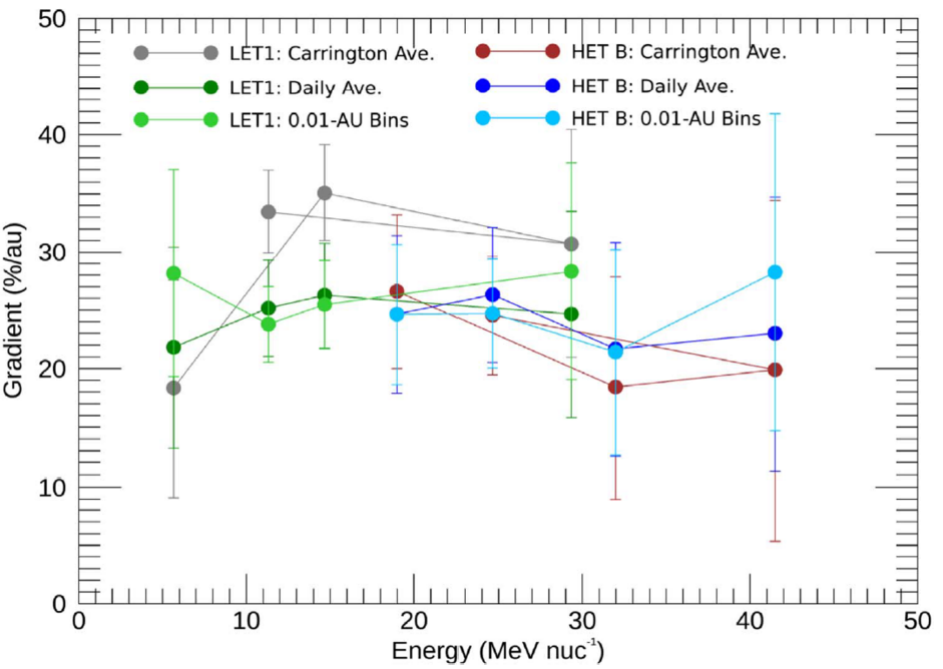
Radial gradients of PSP/ISOIS helium as a function of energy. Data were taken from $\sim2018.7$ to $\sim2019.9$ using the LET1 and HET B telescopes on the EPI-Hi sensor. After subtracting solar energetic particle events and de-trending from long-term changes in solar modulation (using SOHO/EPHIN as a 1-AU baseline; see Fig. 10a), fits were applied to 3 forms of data: (i) averaged over Carrington longitudes in the spacecraft frame (Carrington Ave.), (ii) averaged over daily time scales (Daily Ave.), and (iii) averaged in 0.01 AU radial increments (0.01-AU bins). The results for (ii) and (iii) are mostly consistent with each other, while a possibly larger gradient is evident in (i), for which variations caused by solar wind streams have been minimized. The fluxes used in this analysis reflect the combined contribution from all sources at a given energy (e.g., ACRs dominate but the GCRs have not yet been subtracted). Figure from Rankin et al. (2021)

The first studies of cosmic rays on Parker Solar Probe were conducted by Rankin et al. (2021) and Rankin et al. (2022) who analyzed ACR helium and oxygen into 0.17 and 0.096 AU, respectively, using measurements made by the EPI-Hi sensor of the ISOIS instrument suite (McComas et al. 2016). The resulting event-subtracted spectra are shown in Fig. 10. Like Marquardt et al. (2018), they found that the gradients inside 1 AU were much larger than those at 1 AU and beyond. For example, Rankin et al. (2021) reported a helium radial intensity gradient of $25\pm5\%~\text{AU}^{-1}$ in the $\sim4$ to $\sim45~\text{MeV/nuc}$ energy range, vs. $\sim10\%~\text{AU}^{-1}$ in the inner heliosphere under similar solar cycle conditions (see their Table 1). They also determined the gradient’s magnitude for specific discrete energy intervals, as shown in Fig. 11. Results were acquired by performing log fits in linear space, assuming a differential radial gradient of the form:

3 $$ g_{r} = \frac{1}{f}\frac{\partial f}{\partial r} = \frac{\partial \ln f}{\partial r}. $$

Distinguishing amongst the different populations (i.e., SEPs, ACRs, and GCRs) is challenging. To differentiate between SEPs and cosmic rays, most studies utilize data taken during times of low, or quiet solar activity, ideally during sunspot minimum when cosmic ray fluxes are high. Distinguishing ACRs from GCRs often proves more challenging as many cosmic ray instruments do not distinguish amongst charge states. When their spectra are well defined, as evidenced in Oxygen (Fig. 10b), the two populations can be readily separated by energy. However, for species such as Helium (Fig. 10a), the distinction is not as clear. These results reflect the combination of ACRs and GCRs over their stated energy ranges. Rankin et al. (2021) additionally evaluated the behavior of the gradients of ACRs after GCR subtraction. Using the HELMOD model at 1 AU (Bobik et al. 2012; Boschini et al. 2020, version 4.0.1, 2021 January; www.helmod.org) to approximate the near-Sun behavior of GCRs, they reported a dramatic increase in the magnitude of the ACR gradient: from $25.2\pm4.1\%~\text{AU}^{-1}$ to $34.3\pm5.6\%~\text{AU}^{-1}$ in the 4.0 to 32 MeV/nuc energy range, and $26.3\pm5.8\%~\text{AU}^{-1}$ to $44.7\pm10.2\%~\text{AU}^{-1}$ in the 13 to 45 MeV/nuc range, after GCR background subtraction.

The ACR Oxygen radial gradient reported recently by Rankin et al. (in press) was even larger in magnitude: $49.4\pm8.0\%~\text{AU}^{-1}$ (6.7 to $27~\text{MeV}\,\text{nuc}^{-1}$; GCR’s subtracted and averaged in 0.025-AU radial bins), and showed surprising agreement with the comparable-energy value obtained by Helios $\sim45$ years ago (Marquardt et al. 2018), indicative of a constant radial gradient from 0.1 to 1 AU. For a summary of ACR Oxygen radial gradients measured over the past 5 solar cycles, the reader is referred to Fig. 4 from Rankin et al. (2022).

**Fig. 12:**
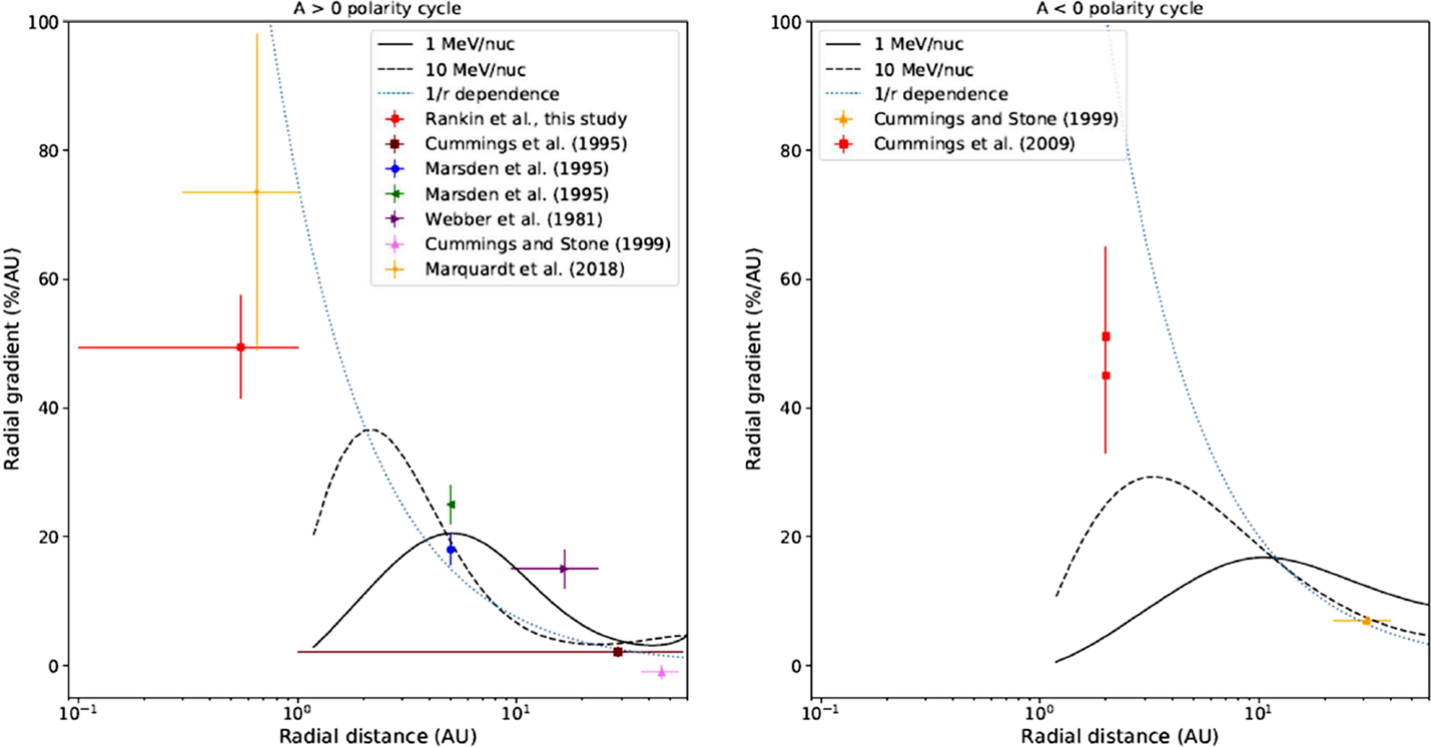
Comparison of simulated and observed radial gradients of ACR Oxygen for $qA>0$ (left) and $qA<0$ (right) polarity cycles. Model results from Strauss and Potgieter (2010) are included, demonstrating clear challenges for reproducing measured gradients inside $\sim2~\text{AU}$. Interestingly, results near the Sun reported by Rankin et al. (2021) and Marquardt et al. (2018) appear more consistent with a $r^{-1}$ behavior rather than that of the standard Parker Spiral assumed here. Figure from Rankin et al. (2022)

Figure 12 presents a comparison of the observed ACR Oxygen gradients during multiple solar minima to results produced by an updated version of the model by Strauss and Potgieter (2010). Beyond $\sim2~\text{AU}$, the model and observations show strong agreement. However, inside $\sim2~\text{AU}$, they start to diverge. This was noted above. While the model predicts a significant decrease toward the Sun, following the typical $r^{-2}$ Parker Spiral behavior, the observations appear more consistent with $\sim r^{-1}$ (as seen in blue dotted line in the left panel of Fig. 12). This and the remarkable consistency between Parker Solar Probe and HELIOS data led Rankin et al. (2022) to suggest a potential explanation: transverse magnetic fluctuations, whose intensity decays as $\sim r^{-1}$, could potentially overwhelm the radial component near the Sun, which decays according to $\sim r^{-2}$, and alter the structure of the magnetic field at scales from $\sim0.1$ to several AU, in such a way as to significantly influence the transport of cosmic rays (e.g. Fisk and Schwadron 1995; Jokipii 2001).

**Fig. 13:**
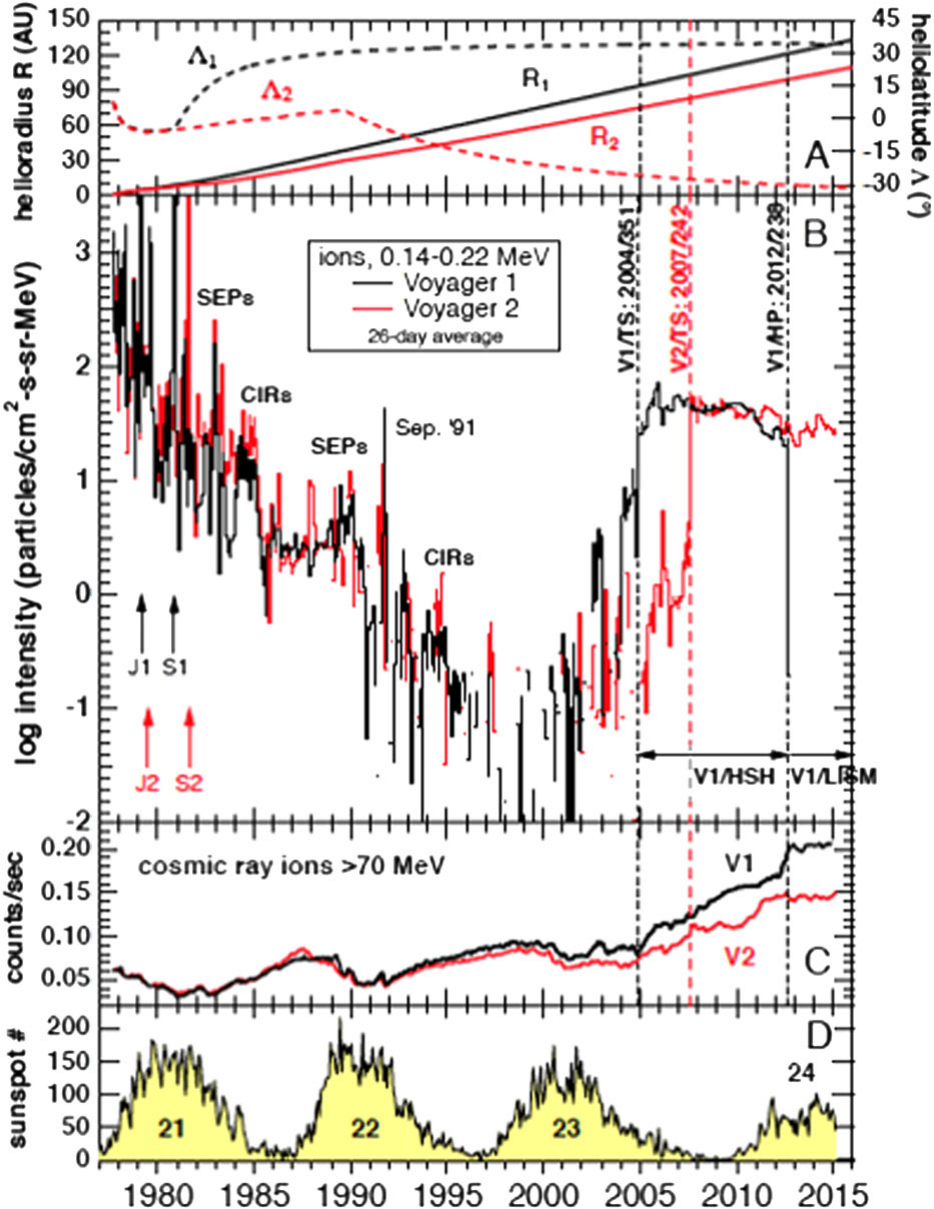
(**A**) Radial distance (solid) and latitude (dashed) of V1 (black) and V2 (red); (**B**) flux of 140–220 keV ions, (**C**) flux of $>270~\text{MeV}$ ions, and (**D**) average sunspot number. This figure was intended for the paper Krimigis and Decker (2015), but only panel B made it to the final publication. It is reproduced here with permission from R. Decker (private communication)

Overall, the new ACR data inside 1 AU presents an intriguing challenge for theorists and modelers investigating particle transport near the Sun. We suggest the interested reader also see Englebrecht et al. (this journal), for more details and recent advances in ACR, GCR, and SEP transport modeling.

## Is the Term “Anomalous” Still Appropriate?

Given that we have learned much about ACRs, it is reasonable to address the question of whether the term “Anomalous Cosmic Ray” is useful. On the one hand, ACRs has become a well known acronym in the heliophysics community, and anomalous cosmic rays are described in many articles and textbooks. Thus, it might cause considerable confusion to introduce a new term. However, to those not familiar with the physics of the outer heliosphere, the term “anomalous” is itself confusing, and not particularly descriptive on its own. By its strict definition, it implies that they are not understood or different from expectations. On the other hand, it is widely accepted that ACRs are interstellar pickup ions that are accelerated in the outer heliosphere. So, they are clearly not anomalous in this context. Moreover, the term “cosmic ray” could cause a bit of confusion to the non-expert as well. Most definitions of this include that the particle move at near the speed of light. However, ACRs generally move only a fraction of the speed of light ($\sim0.4c$ for a 300 MeV proton). Moreover, ACRs are not even the most-energetic species produced in the solar system since the Sun occasionally creates particles with energies of a few GeV, which is more than that of ACRs. The term “solar cosmic ray” was in use some decades ago, but is not as commonly used today.

It should be noted that despite all we have learned about ACRs over the past (nearly) 50 years, there remain important puzzles, particularly concerning the mechanism of acceleration, that are useful to discuss with regards to a consideration of their designation. Prior to the Voyager crossings of the termination shock in 2004 (Voyager 1) and 2007 (Voyager 2), pickup-ion acceleration at the solar-wind termination shock was the paradigm for ACRs. Observations by interplanetary spacecraft prior to this agreed well with the predictions of the theory. The paradigm was challenged when the ACR spectrum did not “unfold”, as expected, when these spacecraft crossed the termination shock (Stone et al. 2005, 2008). However, it is important to note that charged particles with energies $<1~\text{MeV}$ did indeed reach a maximum near the time of the termination shock crossing (Decker et al. 2005, 2008). In fact, they were also observed to be enhanced well before the shock crossing, as can be seen in Fig. 2. Panel (B) of this figure shows the flux of 140–220 keV ions observed by the Voyager LECP experiment for the entire mission since the launch of each spacecraft. Voyager 1 (V1) is in black, and Voyager 2 (V2) in red. The top panel (A) shows the radial location and latitude of each spacecraft, panel (C) shows the flux of $>270~\text{MeV}$ ions, and the bottom panel (D) shows the average sunspot number. The decline in the intensity of 140–220 keV ions after launch is very noteworthy. The dominant source of these particles, during this period, are solar-related energetic particles and their intensity decreases with distance from the Sun. Starting in about 2002, the flux of particles in this energy range began to rise again, reaching a peak at the crossing of the termination shock in late 2004 by Voyager 1 and mid 2007 by Voyager 2, as can be seen in this figure. These particles are known as Termination Shock Particles (TSPs), and are the are likely progenitors of ACRs, as are interstellar pickup ions.

A potentially useful analogy is the acronym “SEP”, for solar energetic particles. SEP is a general name given to energetic particles related to processes occurring at the Sun, including those associated with solar flares, coronal mass ejections (CME), corotating (or stream) interaction regions, etc. In the case of CME-related SEP events, which are among the most intense and reach the highest energies, it is well understood that the shock driven by the CME accelerates the particles. However, only at lower energies is it common to see an intensity peak at the time of arrival of the shock. At energies above a few MeV, the peak is usually well before the arrival of the shock. Scientists call the ones that peak at the shock ESPs (Energetic Storm Particles). These particles are locally accelerated. We do not have a commonly used name for the high-energy ones that do not peak at the shock when it crosses 1 AU. Yet, they are all called SEPs. This is very analogous to the case of acceleration at the termination shock, as described above. The high-energy part, what we call ACRs, did not peak at the shock, but the low-energy part, what we call TSPs, did. Thus, it is reasonable to refer to them all as heliospheric energetic particles, or HEPs. We note that this suggestion also appeared in the article by Wimmer-Schweingruber and Bochsler (2001).

The acronym HEP is to be understood as a general term which includes anomalous cosmic rays and termination shock particles, and possibly others. More specifically, they are energetic particles accelerated in the heliosphere, away from the Sun, and are understood to be distinctly different from SEPs. Interstellar pickup ions could reasonably be included with this general term because they represent a suprathermal population, distinctly different from the solar wind thermal population. Neutral species might also be considered in the general definition since the term “particle” does not restrict it to ionized species. In fact, the Sun produces both energetic neutral atoms and energetic neutrons, which are sometimes generally referred to as SEPs. By extension, one might also consider energetic neutral atoms produced by the processes in the heliosphere to be also included in the general HEP term.

## Summary

We have presented a review of selected topics in the observation, theory, and modeling of anomalous cosmic rays. We reviewed a number of topical subjects, focusing on recent advances in our understanding of the origin and distribution of these particles in the heliosphere, and even their escape. Certainly we were unable to cover all aspects of this topic; however, we have a very extensive list of references at the end of this chapter, which provides the interested reader considerably more sources of information.

We also raised the question of whether the term “anomalous” is still appropriate in describing these particles. Although it is challenging to introduce a new term once it is well established, as ACRs are in this case, we noted that we have learned much about these particles to the point that this term is no longer descriptive or useful, particularly to the broader scientific community. We introduced the term “heliospheric energetic particles” (HEP), of which ACRs are a subset. HEPs represent any suprathermal particle species which originate in the heliosphere, away from the Sun. The latter qualifier is to distinguish them from solar energetic particles, or SEPs. HEPs might reasonably include interstellar pickup ions, in addition to termination shock particles, and ACRs, but exclude GCRs which originate far from the heliosphere. ACRs are the high-energy component of HEPs, while termination shock particles, and interstellar pickup ions are lower-energy components. We suggest that this more-general term is more appropriate, and descriptive to a broader audience, given what we have learned about energetic particles in the heliosphere from nearly five decades of research.

## References

[CR1] Adams J.J.H., Leising M.D. (1991). Maximum distance to the acceleration site of the anomalous component of cosmic rays. International Cosmic Ray Conference.

[CR2] Adams J.J.H., Garcia-Munoz M., Grigorev N.L., Klecker B., Kondratyeve M.A., Mason G.M., McGuire R.E., Mewaldt R.A., Panasyuk M.I., Tretyakova C.A., Tylka A.J., Zhuravlev D.A. (1991). The mean charge state of anomalous cosmic ray oxygen. International Cosmic Ray Conference.

[CR3] Armstrong T.P., Pesses M.E., Decker R.B. (1985). Shock Drift Acceleration.

[CR4] Axford W., Leer E., Skadron G. (1977). The acceleration of cosmic rays by shock waves. Proc 15th ICRC.

[CR5] Barghouty A.F., Jokipii J.R., Mewaldt R.A., Mewaldt R.A., Jokipii J.R., Lee M.A., Möbius E., Zurbuchen T.H. (2000). The transition from singly to multiply-charged anomalous cosmic rays: simulation and interpretation of SAMPEX observations. Acceleration and Transport of Energetic Particles Observed in the Heliosphere.

[CR6] Bell A.R. (1978). The acceleration of cosmic rays in shock fronts. I. Mon. Not. R. Astron. Soc..

[CR7] Blandford R.D., Ostriker J.P. (1978). Particle acceleration by astrophysical shocks. Astrophys. J..

[CR8] Bobik P., Boella G., Boschini M.J., Consolandi C., Torre S.D., Gervasi M., Grandi D., Kudela K., Pensotti S., Rancoita P.G., Tacconi M. (2012). Systematic investigation of solar modulation of galactic protons for solar cycle 23 using a Monte Carlo approach with particle drift effects and latitudinal dependence. Astrophys. J..

[CR9] Boschini M.J., Della Torre S., Gervasi M., Grandi D., Jóhannesson G., La Vacca G., Masi N., Moskalenko I.V., Pensotti S., Porter T.A., Quadrani L., Rancoita P.G., Rozza D., Tacconi M. (2020). Inference of the local interstellar spectra of cosmic-ray nuclei $Z\leq28$ with the GALPROP-HELMOD framework. Astrophys. J. Suppl. Ser..

[CR10] Chalov S.V., Fahr H.J. (1996). Reflection of pre-accelerated pick-up ions at the solar wind termination shock: the seed for anomalous cosmic rays. Sol. Phys..

[CR11] Christon S.P., Cummings A.C., Stone E.C., Behannon K.W., Burlaga L.F., Jokipii J.R., Kota J. (1986). Differential measurement and model calculations of cosmic ray latitudinal gradient with respect to the heliospheric current sheet. J. Geophys. Res..

[CR12] Cummings A.C., Stone E.C., Ormes J.F. (2013). Anomalous cosmic rays. Centenary Symposium 2012: Discovery of Cosmic Rays.

[CR13] Cummings A.C., Stone E.C., Webber W.R. (1987). Latitudinal and radial gradients of anomalous and galactic cosmic rays in the outer heliosphere. Geophys. Res. Lett..

[CR14] Cummings A.C., Stone E.C., McDonald F.B., Heikkila B.C., Lal N., Webber W.R., Heerikhuisen J., Florinski V., Zank G.P., Pogorelov N.V. (2006). Termination shock particle spectral features. Physics of the Inner Heliosheath.

[CR15] Cummings A.C., Stone E.C., McDonald F.B., Heikkila B.C., Lal N., Webber W.R. (2008). Anomalous cosmic rays in the heliosheath. AIP Conf. Proc..

[CR16] Cummings A.C., Tranquille C., Marsden R.G., Mewaldt R.A., Stone E.C. (2009). Radial and latitudinal gradients of anomalous cosmic ray oxygen in the inner heliosphere. Geophys. Res. Lett..

[CR17] Cummings A., Stone E., Heikkila B.C., Lal N., Richardson J. (2019). Voyager 2 observations of the anisotropy of anomalous cosmic rays in the heliosheath. 36th International Cosmic Ray Conference (ICRC2019).

[CR18] Czechowski A., Fichtner H., Grzedzielski S., Hilchenbach M., Hsieh K.C., Jokipii J.R., Kausch T., Kota J., Shaw A. (2001). Anomalous cosmic rays and the generation of energetic neutrals in the region beyond the termination shock. Astron. Astrophys..

[CR19] Decker R.B., Krimigis S.M., Roelof E.C., Hill M.E., Armstrong T.P., Gloeckler G., Hamilton D.C., Lanzerotti L.J. (2005). Voyager 1 in the foreshock, termination shock, and heliosheath. Science.

[CR20] Decker R.B., Krimigis S.M., Roelof E.C., Hill M.E., Armstrong T.P., Gloeckler G., Hamilton D.C., Lanzerotti L.J. (2008). Mediation of the solar wind termination shock by non-thermal ions. Nature.

[CR21] Drake J.F., Opher M., Swisdak M., Chamoun J.N. (2010). A magnetic reconnection mechanism for the generation of anomalous cosmic rays. Astrophys. J..

[CR22] Drury L.O. (1983). An introduction to the theory of diffusive shock acceleration of energetic particles in tenuous plasmas. Rep. Prog. Phys..

[CR23] Dworsky A., Fahr H.J. (2000). Ion acceleration in connection with a modulated solar wind termination shock: phase space propagation and complete energy spectra. Astron. Astrophys..

[CR24] Ellison D.C., Eichler D. (1984). Monte Carlo shock-like solutions to the Boltzmann equation with collective scattering. Astrophys. J..

[CR25] Ellison D.C., Baring M.G., Jones F.C. (1995). Acceleration rates and injection efficiencies in oblique shocks. Astrophys. J..

[CR26] Fahr H.J., Verscharen D. (2008). Ion reflections from the parallel MHD termination shock and a possible injection mechanism into the Fermi-1 acceleration. Astron. Astrophys..

[CR27] Fahr H.J., Sylla A., Fichtner H., Scherer K. (2016). On the evolution of the $\kappa $ distribution of protons in the inner heliosheath. J. Geophys. Res. Space Phys..

[CR28] Ferreira S.E.S., Potgieter M.S., Scherer K. (2007). Transport and acceleration of anomalous cosmic rays in the inner heliosheath. J. Geophys. Res. Space Phys..

[CR29] Fisk L.A., Gloeckler G. (2009). The acceleration of anomalous cosmic rays by stochastic acceleration in the heliosheath. Adv. Space Res..

[CR30] Fisk L.A., Schwadron N.A. (1995). The influence of intermediate-scale variations in the heliospheric magnetic field on the transport of galactic cosmic rays. J. Geophys. Res..

[CR31] Fisk L.A., Kozlovsky B., Ramaty R. (1974). An interpretation of the observed oxygen and nitrogen enhancements in low-energy cosmic rays. Astrophys. J. Lett..

[CR32] Florinski V. (2015). Magnetic flux tube interchange at the heliopause. Astrophys. J..

[CR33] Florinski V., Zank G.P., Jokipii J.R., Stone E.C., Cummings A.C. (2004). Do anomalous cosmic rays modify the termination shock?. Astrophys. J..

[CR34] Florinski V., Decker R.B., le Roux J.A., Zank G.P. (2009). An energetic-particle-mediated termination shock observed by Voyager 2. Geophys. Res. Lett..

[CR35] Florinski V., Jokipii J.R., Alouani-Bibi F., le Roux J.A. (2013). Energetic particle anisotropies at the heliospheric boundary. Astrophys. J. Lett..

[CR36] Florinski V., Stone E.C., Cummings A.C., le Roux J.A. (2015). Energetic particle anisotropies at the heliospheric boundary. II. Transient features and rigidity dependence. Astrophys. J..

[CR37] Frisch P.C. (1996). LISM structure – fragmented superbubble shell?. Space Sci. Rev..

[CR38] Garcia-Munoz M., Mason G.M., Simpson J.A. (1973). The anomalous 1972 low energy galactic cosmic ray proton and helium spectra. International Cosmic Ray Conference.

[CR39] Giacalone J. (2005). Particle acceleration at shocks moving through an irregular magnetic field. Astrophys. J..

[CR40] Giacalone J. (2005). The efficient acceleration of thermal protons by perpendicular shocks. Astrophys. J..

[CR41] Giacalone J., Burgess D. (2010). Interaction between inclined current sheets and the heliospheric termination shock. Geophys. Res. Lett..

[CR42] Giacalone J., Decker R. (2010). The origin of low-energy anomalous cosmic rays at the solar-wind termination shock. Astrophys. J..

[CR43] Giacalone J., Jokipii J.R. (2006). Shock acceleration of high-energy cosmic rays: the importance of the magnetic-field angle. J. Phys. Conf. Ser..

[CR44] Giacalone J., Burgess D., Schwartz S.J., Ellison D.C. (1992). Hybrid simulations of protons strongly accelerated by a parallel collisionless shock. Geophys. Res. Lett..

[CR45] Giacalone J., Drake J.F., Jokipii J.R. (2012). The acceleration mechanism of anomalous cosmic rays. Space Sci. Rev..

[CR46] Giacalone J., Nakanotani M., Zank G.P., Kòta J., Opher M., Richardson J.D. (2021). Hybrid simulations of interstellar pickup protons accelerated at the solar-wind termination shock at multiple locations. Astrophys. J..

[CR47] Gloeckler G., Fisk L.A. (2016). Energetic neutral hydrogen observations demonstrate that Voyager 1 is not observing the extraordinarily strong interstellar magnetic field. Astrophys. J..

[CR48] Guo F., Jokipii J.R., Kota J. (2010). Particle acceleration by collisionless shocks containing large-scale magnetic-field variations. Astrophys. J..

[CR49] Guo X., Florinski V., Wang C. (2018). Effects of anomalous cosmic rays on the structure of the outer heliosphere. Astrophys. J..

[CR50] Guo X., Florinski V., Wang C. (2019). A global MHD simulation of outer heliosphere including anomalous cosmic-rays. Astrophys. J..

[CR51] Heber B. (2001). Modulation of galactic and anomalous cosmic rays in the inner heliosphere. Adv. Space Res..

[CR52] Heber B., Keppler E., Fraenz M., Kunow H. (1999). Variations of anomalous and galactic cosmic ray fluxes in the northern hemisphere: ULYSSES EPAC and KET observations. 26th International Cosmic Ray Conference (ICRC26).

[CR53] Hill M.E., Hamilton D.C., Krimigis S.M. (2002). Evolution of anomalous cosmic-ray oxygen and helium energy spectra during the solar cycle 22 recovery phase in the outer heliosphere. Astrophys. J. Lett..

[CR54] Hovestadt D., Vollmer O., Gloeckler G., Fan C.Y. (1973). Differential energy spectra of low-energy ($<8.5~\text{MeV}$ per nucleon) heavy cosmic rays during solar quiet times. Geophys. Res. Lett..

[CR55] Izmodenov V.V., Alexashov D.B. (2020). Magnitude and direction of the local interstellar magnetic field inferred from Voyager 1 and 2 interstellar data and global heliospheric model. Astron. Astrophys..

[CR56] Jokipii J.R. (1986). Particle acceleration at a termination shock 1. Application to the solar wind and the anomalous component. J. Geophys. Res..

[CR57] Jokipii J.R., Jones W.V., Kerr F.J., Ormes J.F. (1990). Cosmic rays in the heliosphere: present status and future opportunities. Particle Astrophysics – the NASA Cosmic Ray Program for the 1990s and Beyond.

[CR58] Jokipii J.R. (1992). Constraints on the acceleration of anomalous cosmic rays. Astrophys. J. Lett..

[CR59] Jokipii J.R. (1996). Theory of multiply charged anomalous cosmic rays. Astrophys. J. Lett..

[CR60] Jokipii J.R. (2001). Latitudinal heliospheric magnetic field: stochastic and causal components. J. Geophys. Res. Space Phys..

[CR61] Jokipii J.R., Giacalone J. (1998). The theory of anomalous cosmic rays. Space Sci. Rev..

[CR62] Jokipii J.R., Kota J. (1989). The polar heliospheric magnetic field. Geophys. Res. Lett..

[CR63] Jokipii J.R., Kota J. (2001). Cosmic-ray effects of propagating shocks including the heliosheath. International Cosmic Ray Conference.

[CR64] Jokipii J.R., Thomas B. (1981). Effects of drift on the transport of cosmic rays. IV – modulation by a wavy interplanetary current sheet. Astrophys. J..

[CR65] Jokipii J.R., Giacalone J., Kóta J. (2004). Transverse streaming anisotropies of charged particles accelerated at the solar wind termination shock. Astrophys. J. Lett..

[CR66] Kallenbach R., Bamert K., Hilchenbach M. (2009). Acceleration of the anomalous component of cosmic rays revisited. Astrophys. Space Sci. Trans..

[CR67] Keppler E., Blake J.B., Hovestadt D., Korth A., Quenby J., Umlauft G., Woch J. (1992). The ULYSSES energetic particle composition experiment EPAC. Astron. Astrophys. Suppl. Ser..

[CR68] Klecker B. (1995). The anomalous component of cosmic rays in the 3-D heliosphere. Space Sci. Rev..

[CR69] Klecker B., Hovestadt D., Gloeckler G., Fan C.Y. (1980). On the charge state of the anomalous oxygen component. Geophys. Res. Lett..

[CR70] Klecker B., Hovestadt D., Scholer M., Arbinger H., Ertl M., Kaestle H., Kuenneth E., Laeverenz P., Seidenschwang E., Blake J.B. (1993). HILT – a heavy ion large area proportional counter telescope for solar and anomalous cosmic rays. IEEE Trans. Geosci. Remote Sens..

[CR71] Klecker B., Oetliker M., Blake J.B., Hovestadt D., Mason G.M., Mazur J.E., McNab M.C. (1997). Multiply charged anomalous cosmic ray N, O and Ne: observations with HILT/SAMPEX. International Cosmic Ray Conference.

[CR72] Kóta J. (2010). Particle acceleration at near-perpendicular shocks: the role of field-line topology. Astrophys. J..

[CR73] Kota J., Jokipii J.R. (1983). Effects of drift on the transport of cosmic rays. VI – a three-dimensional model including diffusion. Astrophys. J..

[CR74] Kóta J., Jokipii J.R. (2008). Anomalous cosmic rays in the heliosheath: simulation with a blunt termination shock. AIP Conf. Proc..

[CR75] Krimigis S.M., Decker R.B. (2015). The Voyagers’ odyssey. Am. Sci..

[CR76] Krimigis S.M., Decker R.B., Hill M.E., Armstrong T.P., Gloeckler G., Hamilton D.C., Lanzerotti L.J., Roelof E.C. (2003). Voyager 1 exited the solar wind at a distance of $\sim85~\text{AU}$ from the Sun. Nature.

[CR77] Krimigis S.M., Decker R.B., Roelof E.C., Hill M.E., Armstrong T.P., Gloeckler G., Hamilton D.C., Lanzerotti L.J. (2013). Search for the exit: Voyager 1 at heliosphere’s border with the galaxy. Science.

[CR78] Krimigis S.M., Decker R.B., Roelof E.C., Hill M.E., Bostrom C.O., Dialynas K., Gloeckler G., Hamilton D.C., Keath E.P., Lanzerotti L.J. (2019). Energetic charged particle measurements from Voyager 2 at the heliopause and beyond. Nat. Astron..

[CR79] Krymsky G.F. (1977). A regular mechanism for the acceleration of charged particles on the front of a shock wave. Dokl. Akad. Nauk SSSR.

[CR80] Langner U.W., Potgieter M.S., Fichtner H., Borrmann T. (2006). Modulation of anomalous protons: effects of different solar wind speed profiles in the heliosheath. J. Geophys. Res. Space Phys..

[CR81] Lanzerotti L.J., Maclennan C.G. (1995). Anomalous cosmic ray oxygen and neon ($\sim2.4~\text{MeV/nucl}$) at high southern heliolatitudes. Geophys. Res. Lett..

[CR82] Lario D., Berger L., Decker R.B., Wimmer-Schweingruber R.F., Wilson I.L.B., Giacalone J., Roelof E.C. (2019). Evolution of the suprathermal proton population at interplanetary shocks. Astron. J..

[CR83] Lazarian A., Opher M. (2009). A model of acceleration of anomalous cosmic rays by reconnection in the heliosheath. Astrophys. J..

[CR84] le Roux J.A., Fichtner H. (1997). A self-consistent determination of the heliospheric termination shock structure in the presence of pickup, anomalous, and galactic cosmic ray protons. J. Geophys. Res..

[CR85] le Roux J.A., Fichtner H. (1999). Global merged interaction regions, the heliospheric termination shock, and time-dependent cosmic ray modulation. J. Geophys. Res..

[CR86] le Roux J.A., Webb G.M. (2009). Time-dependent acceleration of interstellar pickup ions at the heliospheric termination shock using a focused transport approach. Astrophys. J..

[CR87] le Roux J.A., Fichtner H., Zank G.P. (2000). Self-consistent acceleration of multiply reflected pickup ions at a quasi-perpendicular solar wind termination shock: a fluid approach. J. Geophys. Res..

[CR88] Leske R.A., Cummings A.C., Mewaldt R.A., Stone E.C. (2013). Anomalous and galactic cosmic rays at 1 AU during the cycle 23/24 solar minimum. Space Sci. Rev..

[CR89] Li G., Zank G.P., Heerikhuisen J., Florinski V., Zank G.P., Pogorelov N.V. (2006). Particle acceleration at a rippling termination shock. Physics of the Inner Heliosheath.

[CR90] Liu S., Jokipii J.R. (2021). Acceleration of charged particles in astrophysical plasmas. Front. Astron. Space Sci..

[CR91] Marquardt J., Heber B., Potgieter M.S., Strauss R.D. (2018). Energy spectra of carbon and oxygen with HELIOS E6. Radial gradients of anomalous cosmic ray oxygen within 1 AU. Astron. Astrophys..

[CR92] Marsden R.G., Sanderson T.R., Tranquille C., Trattner K.J., Anttila A., Torsti J. (1999). On the gradients of ACR oxygen at intermediate heliocentric distances: Ulysses/Soho results. Adv. Space Res..

[CR93] McComas D.J., Schwadron N.A. (2006). An explanation of the Voyager paradox: particle acceleration at a blunt termination shock. Geophys. Res. Lett..

[CR94] McComas D.J., Ebert R.W., Elliott H.A., Goldstein B.E., Gosling J.T., Schwadron N.A., Skoug R.M. (2008). Weaker solar wind from the polar coronal holes and the whole Sun. Geophys. Res. Lett..

[CR95] McComas D.J., Alexander N., Angold N., Bale S., Beebe C., Birdwell B., Boyle M., Burgum J.M., Burnham J.A., Christian E.R., Cook W.R., Cooper S.A., Cummings A.C., Davis A.J., Desai M.I., Dickinson J., Dirks G., Do D.H., Fox N., Giacalone J., Gold R.E., Gurnee R.S., Hayes J.R., Hill M.E., Kasper J.C., Kecman B., Klemic J., Krimigis S.M., Labrador A.W., Layman R.S., Leske R.A., Livi S., Matthaeus W.H., McNutt R.L., Mewaldt R.A., Mitchell D.G., Nelson K.S., Parker C., Rankin J.S., Roelof E.C., Schwadron N.A., Seifert H., Shuman S., Stokes M.R., Stone E.C., Vandegriff J.D., Velli M., von Rosenvinge T.T., Weidner S.E., Wiedenbeck M.E., Wilson P. (2016). Integrated science investigation of the Sun (ISIS): design of the energetic particle investigation. Space Sci. Rev..

[CR96] McComas D.J., Christian E.R., Cohen C.M.S., Cummings A.C., Davis A.J., Desai M.I., Giacalone J., Hill M.E., Joyce C.J., Krimigis S.M., Labrador A.W., Leske R.A., Malandraki O., Matthaeus W.H., McNutt R.L., Mewaldt R.A., Mitchell D.G., Posner A., Rankin J.S., Roelof E.C., Schwadron N.A., Stone E.C., Szalay J.R., Wiedenbeck M.E., Bale S.D., Kasper J.C., Case A.W., Korreck K.E., MacDowall R.J., Pulupa M., Stevens M.L., Rouillard A.P. (2019). Probing the energetic particle environment near the Sun. Nature.

[CR97] McDonald F.B., Teegarden B.J., Trainor J.H., Webber W.R. (1974). The anomalous abundance of cosmic-ray nitrogen and oxygen nuclei at low energies. Astrophys. J. Lett..

[CR98] McDonald F.B., Heikkila B., Lal N., Stone E.C. (2000). The relative recovery of galactic and anomalous cosmic rays in the distant heliosphere: evidence for modulation in the heliosheath. J. Geophys. Res..

[CR99] McKibben R.B., Pyle K.R., Simpson J.A. (1979). The solar latitude and radial dependence of the anomalous cosmic-ray helium component. Astrophys. J. Lett..

[CR100] Mewaldt R.A., Heerikhuisen J., Florinski V., Zank G.P., Pogorelov N.V. (2006). Implications of multiply-charged anomalous cosmic rays. Physics of the Inner Heliosheath.

[CR101] Mewaldt R.A., Cummings A.C., Cummings J.R., Stone E.C., Klecker B., Hovestadt D., Scholer M., Mason G.M., Mazur J.E., von Hamilton D.C., Rosenvinge T.T., Blake J.B. (1993). The return of the anomalous cosmic rays to 1 AU in 1992. Geophys. Res. Lett..

[CR102] Mewaldt R.A., Selesnick R.S., Cummings J.R., Stone E.C., von Rosenvinge T.T. (1996). Evidence for multiply charged anomalous cosmic rays. Astrophys. J. Lett..

[CR103] Mewaldt R.A., Davis A.J., Lave K.A., Leske R.A., Stone E.C., Wiedenbeck M.E., Binns W.R., Christian E.R., Cummings A.C., de Nolfo G.A., Israel M.H., Labrador A.W., von Rosenvinge T.T. (2010). Record-setting cosmic-ray intensities in 2009 and 2010. Astrophys. J. Lett..

[CR104] Moraal H., Stoker P.H. (2010). Long-term neutron monitor observations and the 2009 cosmic ray maximum. J. Geophys. Res. Space Phys..

[CR105] Moraal H., Caballero-Lopez R.A., McCracken K.G., McDonald F.B., Mewaldt R.A., Ptuskin V., Wiedenbeck M.E., Heerikhuisen J., Florinski V., Zank G.P., Pogorelov N.V. (2006). Cosmic ray energy changes at the termination shock and in the heliosheath. Physics of the Inner Heliosheath.

[CR106] Neergaard-Parker L., Zank G.P. (2012). Particle acceleration at quasi-parallel shock waves: theory and observations at 1 AU. Astrophys. J..

[CR107] Neergaard-Parker L., Zank G.P., Hu Q. (2014). Particle acceleration at quasi-perpendicular shock waves: theory and observations at 1 AU. Astrophys. J..

[CR108] Parker E.N. (1965). The passage of energetic charged particles through interplanetary space. Planet. Space Sci..

[CR109] Pesses M.E., Jokipii J.R., Eichler D. (1981). Cosmic ray drift, shock wave acceleration, and the anomalous component of cosmic rays. Astrophys. J. Lett..

[CR110] Potgieter M.S., Moraal H. (1988). Acceleration of cosmic rays in the solar wind termination shock. I. A steady state technique in a spherically symmetric model. Astrophys. J..

[CR111] Rankin J.S., McComas D.J., Leske R.A., Christian E.R., Cohen C.M.S., Cummings A.C., Joyce C.J., Labrador A.W., Mewaldt R.A., Posner A., Schwadron N.A., Strauss R.D., Stone E.C., Wiedenbeck M.E. (2021). First observations of anomalous cosmic rays in to 36 solar radii. Astrophys. J..

[CR112] Rankin J.S., McComas D.J., Leske R.A., Christian E.R., Cohen C.M.S., Cummings A.C., Joyce C.J., Labrador A.W., Mewaldt R.A., Schwadron N.A., Stone E.C., Strauss R.D., Wiedenbeck M.E. (2022). Anomalous cosmic-ray oxygen observations into 0.1 au. Astrophys. J..

[CR113] Richardson J.D., Stone E.C., Cummings A.C., Kasper J.C., Zhang M., Burlaga L.F., Ness N.F., Liu Y. (2006). Correlation between energetic ion enhancements and heliospheric current sheet crossings in the outer heliosphere. Geophys. Res. Lett..

[CR114] Scherer K., Fichtner H., Fahr H.J. (1997). The acceleration time of ACR: constraints from Pioneer 10 data. International Cosmic Ray Conference.

[CR115] Scherer K., Ferreira S.E.S., Potgieter M.S., Fichtner H., Heerikhuisen J., Florinski V., Zank G.P., Pogorelov N.V. (2006). Time- and latitude-dependence of the compression ratio and the injection rate at the heliospheric termination shock. Physics of the Inner Heliosheath.

[CR116] Scholer M. (1990). Diffuse ions at a quasi-parallel collisionless shock – simulations. Geophys. Res. Lett..

[CR117] Schwadron N.A., Lee M.A., McComas D.J. (2008). Diffusive acceleration at the blunt termination shock. Astrophys. J..

[CR118] Selesnick R.S., Mewaldt R.A., Cummings J.R. (1997). Multiply charged anomalous cosmic rays above 15 MeV/nucleon. International Cosmic Ray Conference.

[CR119] Senanayake U.K., Florinski V., Cummings A.C., Stone E.C. (2015). Spectral evolution of anomalous cosmic rays at Voyager 1 beyond the termination shock. Astrophys. J..

[CR120] Siewert M., Fahr H.J., McComas D.J., Schwadron N.A. (2013). Spectral properties of keV-energetic ion populations inside the heliopause reflected by IBEX-relevant energetic neutral atoms. Astron. Astrophys..

[CR121] Simpson J.A., Anglin J.D., Balogh A., Bercovitch M., Bouman J.M., Budzinski E.E., Burrows J.R., Carvell R., Connell J.J., Ducros R., Ferrando P., Firth J., Garcia-Munoz M., Henrion J., Hynds R.J., Iwers B., Jacquet R., Kunow H., Lentz G., Marsden R.G., Mckibben R.B., Meuller-Mellin R., Page D.E., Perkins M., Raviart A., Sanderson T.R., Sierks H., Treguer L., Tuzzolino A.J., Wenzel K.P., Wibberenz G. (1992). The ULYSSES cosmic ray and solar particle investigation. Astron. Astrophys. Suppl. Ser..

[CR122] Simpson J.A., Connell J.J., Lopate C., McKibben R.B., Zhang M. (1995). The latitude gradients of galactic cosmic ray and anomalous helium fluxes measured on Ulysses from the Sun’s south polar region to the equator. Geophys. Res. Lett..

[CR123] Simpson J.A., Zhang M., Bame S. (1996). A solar polar north-south asymmetry for cosmic-ray propagation in the heliosphere: the ULYSSES pole-to-pole rapid transit. Astrophys. J. Lett..

[CR124] Smith E.J., Balogh A. (2008). Decrease in heliospheric magnetic flux in this solar minimum: recent Ulysses magnetic field observations. Geophys. Res. Lett..

[CR125] Sokół J.M., Bzowski M., Tokumaru M. (2019). Interstellar neutral gas species and their pickup ions inside the heliospheric termination shock. Ionization rates for H, O, Ne, and He. Astrophys. J..

[CR126] Steenberg C.D., Moraal H. (1996). An acceleration/modulation model for anomalous cosmic-ray hydrogen in the heliosphere. Astrophys. J..

[CR127] Steenberg C.D., Moraal H. (1999). Form of the anomalous cosmic ray spectrum at the solar wind termination shock. J. Geophys. Res..

[CR128] Stone E.C., Cummings A.C., Hamilton D.C., Hill M.E., Krimigis S.M. (1999). Voyager observations of anomalous and galactic cosmic rays during 1998. Proc of the 26th ICRC 7.

[CR129] Stone E.C., Cummings A.C., McDonald F.B., Heikkila B.C., Lal N., Webber W.R. (2005). Voyager 1 explores the termination shock region and the heliosheath beyond. Science.

[CR130] Stone E.C., Cummings A.C., McDonald F.B., Heikkila B.C., Lal N., Webber W.R. (2008). An asymmetric solar wind termination shock. Nature.

[CR131] Stone E.C., Cummings A.C., McDonald F.B., Heikkila B.C., Lal N., Webber W.R. (2013). Voyager 1 observes low-energy galactic cosmic rays in a region depleted of heliospheric ions. Science.

[CR132] Strauss R.D., Fichtner H. (2014). Cosmic ray anisotropies near the heliopause. Astron. Astrophys..

[CR133] Strauss R.D., Potgieter M.S. (2010). Modeling anomalous cosmic ray oxygen gradients over successive solar cycles. J. Geophys. Res. Space Phys..

[CR134] Strauss R.D., Potgieter M.S., Ferreira S.E.S. (2010). The heliospheric transport and modulation of multiple charged anomalous oxygen revisited. Astron. Astrophys..

[CR135] Strauss R.D., Potgieter M.S., Ferreira S.E.S., Hill M.E. (2010). Modelling anomalous cosmic ray oxygen in the heliosheath. Astron. Astrophys..

[CR136] Strauss R.D., le Roux J.A., Engelbrecht N.E., Ruffolo D., Dunzlaff P. (2016). Non-axisymmetric perpendicular diffusion of charged particles and their transport across tangential magnetic discontinuities. Astrophys. J..

[CR137] Trattner K.J., Marsden R.G., Bothmer V., Sanderson T.R., Wenzel K.P., Klecker B., Hovestadt D. (1995). The Ulysses south polar pass: anomalous component of cosmic rays. Geophys. Res. Lett..

[CR138] Trattner K.J., Marsden R.G., Sanderson T.R., Wenzel K.P., Klecker B., Hovestadt D. (1995). The anomalous component of cosmic rays: oxygen latitudinal gradient. Geophys. Res. Lett..

[CR139] Trattner K.J., Marsden R.G., Bothmer V., Sanderson T.R., Wenzel K.P., Klecker B., Hovestadt D. (1996). ULYSSES COSPIN/LET: latitudinal gradients of anomalous cosmic ray O, N and Ne. Astron. Astrophys..

[CR140] Verscharen D., Fahr H.J. (2008). Self-initialised Fermi-1 acceleration by pitch-angle re-scattering of solar wind ions reflected from the parallel termination shock. Astrophys. Space Sci. Trans..

[CR141] Webber W.R., von McDonald F.B., Rosenvinge T.T., Mewaldt R.A. (1981). A study of temporal and radial dependencies of the anomalous helium and oxygen nuclei. International Cosmic Ray Conference.

[CR142] Webber W.R., Lockwood J.A., McDonald F.B., Heikkila B. (2001). Using transient decreases of cosmic rays observed at Voyagers 1 and 2 to estimate the location of the heliospheric termination shock. J. Geophys. Res..

[CR143] Webber W.R., Cummings A.C., McDonald F.B., Stone E.C., Heikkila B., Lal N. (2007). Temporal and spectral variations of anomalous oxygen nuclei measured by Voyager 1 and Voyager 2 in the outer heliosphere. J. Geophys. Res. Space Phys..

[CR144] Wimmer-Schweingruber R.F., Bochsler P., Scherer K., Fichtner H., Fahr H.J., Marsch E. (2001). A non-solar origin of the “SEP” component in lunar soils. The Outer Heliosphere: The Next Frontiers.

[CR145] Zank G.P. (1999). Interaction of the solar wind with the local interstellar medium: a theoretical perspective. Space Sci. Rev..

[CR146] Zank G.P., Webb G.M., Donohue D.J. (1993). Particle injection and the structure of energetic-particle-modified shocks. Astrophys. J..

[CR147] Zhao L.L., Zank G.P., Hu Q., Chen Y., Adhikari L., le Roux J.A., Cummings A., Stone E., Burlaga L.F. (2019). ACR proton acceleration associated with reconnection processes beyond the heliospheric termination shock. Astrophys. J..

[CR148] Zirnstein E.J., Kumar R., Heerikhuisen J., McComas D.J., Galli A. (2018). Stochastic acceleration of $\sim0.1\text{--}5~\text{keV}$ pickup ions in the heliotail. Astrophys. J..

[CR149] Zirnstein E.J., Kumar R., Bandyopadhyay R., Dayeh M.A., Heerikhuisen J., McComas D.J. (2021). Turbulent acceleration of interstellar pickup ions at the heliospheric termination shock forms the global ENA spectrum. Astrophys. J. Lett..

